# Co-detection of mutations and methylations in cerebrospinal fluid ctDNA for minimally-invasive diagnosis of brainstem glioma

**DOI:** 10.1186/s13046-025-03455-y

**Published:** 2025-10-07

**Authors:** Tian Li, Huan Wang, Yujin Wang, Yiying Mai, Pei Wang, Mingxin Zhang, Zhuang Jiang, Luyang Xie, Hang Zhou, Yi Wang, Xiaoou Li, Dan Xiao, Jingyao Geng, Wenhao Wu, Peng Zhang, Liang Wang, Zhen Wu, Junting Zhang, Dandan Cao, Changchun Pan, Liwei Zhang

**Affiliations:** 1 Department of Neurosurgery, Beijing Tiantan Hospital, Capital Medical University, No. 119 Nan Si Huan West Road, Fengtai District, Beijing, China; 2 Beijing Neurosurgical Institute, Beijing, China; 3 China National Clinical Research Center for Neurological Diseases, Beijing Tiantan Hospital, Beijing, 100070 China; 4 Beijing Key Laboratory of Brain Tumor, Beijing Tiantan Hospital, Nan Si Huan West Road 119, Fengtai District, Beijing, 100070 China; 5 Genetron Health (Beijing) Co. Ltd, No. 8 Shengmingyuan Middle Road, Changping District, Beijing, 102206 China

**Keywords:** Brainstem glioma, Diffuse midline glioma, Circulating tumor DNA, DNA methylation, Liquid biopsy

## Abstract

**Background:**

Genetic and epigenetic profiles are critical in managing brainstem gliomas (BSG), whose heterogeneity is far beyond the realm of the Diffuse midline glioma, H3K27 altered. Cerebrospinal fluid (CSF) circulating tumor DNA (ctDNA)-based liquid biopsy provides minimally-invasive strategies to acquire molecular information for brain tumors, whereas there is a deficiency in techniques for co-detection of genetic and epigenetic alterations due to the limited yield of ctDNA. This study aims to develop a reliable minimally-invasive approach to simultaneously detect the mutation and methylation profiles in the CSF ctDNA of BSGs, thereby enhancing diagnostic accuracy, prognostic capability, and monitoring potential.

**Methods:**

A cohort of 80 BSG cases with 138 CSF samples and 71 tissues was retrospectively established. Public tissue methylation profiles (*N* = 1016) were used for the development of H3K27M and IDH mutation-specific assay. The mutation and methylation co-detection classifier (BSGdiag) was trained and tested in tissue cohorts and further validated in CSF samples. CSF Methylation Risk Score (MRS) was defined and used for prognostication and monitoring.

**Results:**

The methylation assay demonstrated robust three-class (H3K27M-mut, IDH-mut and double-wildtype) classification with microAUC values of 1.00, 0.973, and 0.813 across public datasets, tissue cohorts, and CSF samples, respectively. BSGdiag achieved a sensitivity of 95.6%, specificity of 83.3%, and AUC of 0.949 for the H3K27M subtype, and a microAUC of 0.990 for the three-class classification in CSFs. MRS-stratified CSF methylation risk group was an independent prognostic factor (HR = 2.61, 95% CI: 1.09–6.25, *P* = 0.032). Methylation information in CSF remained even with clinical, radiological and CSF genetic indications of no disease, suggesting its utility in monitoring minimal residual disease.

**Conclusions:**

The study de novo developed the first methylation assay for robust BSG molecular subtyping and introduced a novel methodology for co-detecting CSF ctDNA mutations and methylation in BSGs. The BSGdiag enhances the utility of ctDNA by leveraging both genetic and epigenetic information. Its comprehensiveness, minimal invasiveness, robustness, and reliability make it highly promising for future clinical applications and trial designs.

**Supplementary Information:**

The online version contains supplementary material available at 10.1186/s13046-025-03455-y.

## Introduction

The brainstem is essential for life, and gliomas arising from the brainstem (BSG) remain the most formidable challenge for both neurosurgeons and neuro-oncologists. The most recognized BSG, diffuse intrinsic pontine glioma (DIPG), is now classified as diffuse midline glioma, H3K27-altered (DMG, H3K27-alt), along with other diffuse midline gliomas, in the 5th edition of the WHO classification of central nervous tumors (WHO CNS5) [1]. However, increasing evidence illustrates the significant molecular heterogeneity of BSG. Specifically, DIPGs characterized by H3K27 alterations carry the most dismal prognosis with a median overall survival (mOS) of less than 1 year and are resistant to radiotherapies and chemotherapies [2]. On the other hand, IDH-mutated BSG (median OS of 124.8 months) [3] or those with neither H3K27M nor IDH mutations (‘Double-Wildtype’, with a 10-year OS rate of more than 90%)^4^ have distinctively better prognoses.

Besides these driver mutations, other concurrent gene mutations, including TP53, PPM1D, ATRX, BRAF, NF1, FGFR1, and TERT promoter, also bear substantial diagnostic and prognostic significance [5–7]. Furthermore, DNA methylation has shown superior stratification potential compared to gene mutation [1, 8]. We have demonstrated that despite the presence of H3K27M, which has a dominant influence on OS, DNA methylation can further stratify BSG into H3-pons and H3-medulla subclass, with distinctive prognosis and underlying uncharted mechanism [9]. Integrating genetic mutation and DNA methylation profiles can improve diagnostic accuracy, decrease inconclusive diagnoses, and facilitate prognostic subclassification [10–14], which is recommended by guidelines such as WHO CNS5, NCCN, and EANO in various countries and regions [1, 15, 16].

Furthermore, current treatments, including surgery, radiotherapy, novel pharmaceuticals like ONC201/206, IDH mutation inhibitors, and clinical trials involving immune therapies are all reliant on molecular data [17–21]. Therefore, molecular profiling of BSG is essential for accurate diagnosis and management. However, due to the eloquent location of the brainstem, both craniotomy and stereotactic biopsy pose substantial risks, and tumor tissue-based sequencing is susceptible to sampling bias, potentially precluding and/or misguiding further therapies. Moreover, these invasive procedures cannot be performed repeatedly. Liquid biopsy is a promising non-invasive approach for molecular profiling of BSG.

In recent years, cerebrospinal fluid (CSF) has emerged as a valuable source for detecting tumor-specific genetic mutations through circulation tumor DNA (ctDNA). Next-generation sequencing (NGS) provides a comprehensive approach, with sensitivity for glioma-related mutations ranging from 49.7–82.5% [22, 23]. In contrast, digital droplet PCR (ddPCR) is effective for targeting one or a few specific mutations, such as H3K27M, enhancing sensitivity reached from 87–96.5% [24, 25].

Nonetheless, the substitution of tissue biopsies with CSFs for diagnostic purposes still encounters several significant challenges, including diminished sensitivity and inadequate detection of tumor-specific alterations. These issues can elevate the risk of erroneous clinical decisions and impede the broader adoption of CSF-based diagnostic methods in clinical practice. Despite the advantages of combined gene mutation and DNA methylation analysis on tumor tissue, currently, to our knowledge, no studies have validated the use of CSF ctDNA for detecting BSG/DIPG/DMG-specific methylation features. This is especially challenging due to the low yield of CSF ctDNA, which frequently not suffice to meet the threshold of 20-250ng required for genome-wide methylation profiling utilizing 450/850K methylation arrays [16, 26, 27]. More importantly, to date, no studies in the field of brain tumors have achieved the integration of methylation and mutation detection in CSFs, much less in the realm of BSGs.

Thus, to overcome the problems faced by tissue or CSF-based molecular profiling of BSG and bring the potential of liquid biopsy closer to practical, real-world application, here we developed and validated a novel methylation assay specific to BSGs and constructed a diagnostic model named BSGdiag, which leveraged both mutation and methylation detection from CSFs, and achieved enhanced diagnostic precision, with a sensitivity of 95.6%, specificity of 83.3% and AUC of 0.949 for the H3K27M subtype, and a micro AUC of 0.990 for the molecular three-class classification, in CSF testing cohort. This study will pave the way for future diagnostic clinical trials.

## Materials and methods

### Study design and sample obtaining

**Fig. 1:**
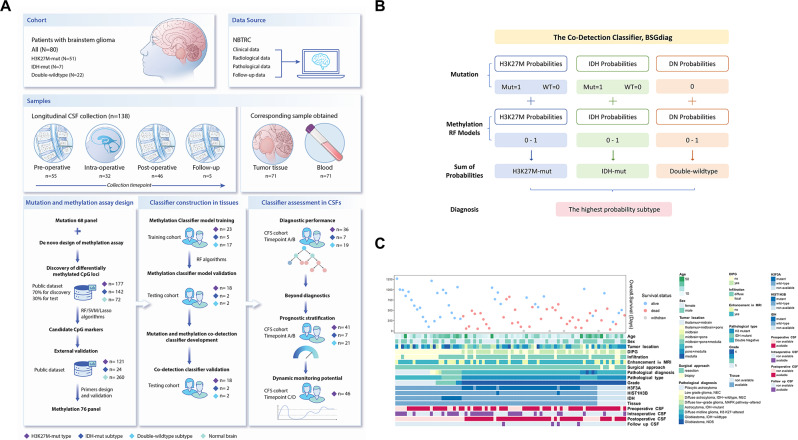
Study design and patient baseline characteristics. **A**. Overview of the study design, outlining cohort composition, sources of clinical data, sample distribution, and sequential research steps. **B**. Diagram illustrating the BSGdiag decision framework, which combines predefined mutation-based probabilities and methylation-derived prediction scores to determine glioma subtypes. For each sample, mutation presence in key driver genes directly sets a subtype-specific probability, while methylation signatures provide complementary probabilistic estimates. The final diagnosis is assigned based on the highest combined probability across the three subtypes. **C**. Heatmap summarizing the baseline clinical and molecular characteristics of all patients included in the study cohort Abbreviations: DIPG, diffuse intrinsic pontine glioma; NBTRC, National Brain Tumor Registry Center of China

The study was approved by the Ethics Committee of Beijing Tiantan Hospital (KY2024-139-01). All patients provided informed consent, and the study adhered to the Declaration of Helsinki. The study design is summarized in Fig. 1A. This study comprised three primary phases: (1) Screening and validating BSG-specific methylation biomarkers using public databases; (2) Training and testing a diagnostic classifier integrating methylation and mutation profiles from tissue samples; (3) Evaluating the classifier’s diagnostic efficacy in CSFs. Additionally, the study explored the prognostic capabilities of methylation and monitored ctDNA dynamics throughout treatment in parallel.

This observational, retrospective study included patients who underwent tumor resection or stereotactic biopsy at Beijing Tiantan Hospital from October 2018 to July 2023. 138 CSF samples from 80 eligible BSG patients were collected. CSF samples (3–10 ml) were collected at multiple timepoints: 54 preoperative (Point A), 32 intraoperative (Point B), 46 postoperative (Point C), and 5 during follow-up (Point D). Additionally, 71 tissue samples were obtained, and paired peripheral blood samples were collected. Details are provided in the Supplementary Methods.

### Clinical, radiological, pathological data and follow-up

The clinical, radiological, pathological, and follow-up parameters were extracted from the National Brain Tumor Registry Center of China (NBTRC) [28, 29]. Pathological diagnosis were re-reviewed by two senior pathologists based on WHO CNS5. Patients with glioma exhibiting positive immunohistochemical staining for H3K27M or mutations in H3F3A or HIST1H3B/C were classified as the H3K27M-mut subtype. Those with positive IDH1/2-mutant staining or sequencing were classified as the IDH-mut subtype, while all the others were defined as the Double-Wildtype (DW) subtype. Overall survival (OS) was defined as the duration from the date of the first MRI to the date of the last follow-up or the patient’s death.

### Mutation and methylation assay design

A panel of 68 glioma-related genes was used for mutation testing, as described in previous research [23]. For the methylation assay design, methylation biomarkers were selected from public datasets, and primers were designed for targeted CpGs (Fig. 1A). Datasets GSE90496 and GSE109379 were accessed to select methylation markers from H3K27M-mut gliomas (*N* = 117), IDH-mut gliomas (*N* = 422), and normal brain tissues (*N* = 72) [9, 10, 30–32]. The dataset was split, with 70% used for discovery, identifying highly differentially methylated CpG loci with Wilcoxon-rank sum test and stringent cutoff of 0.3 delta β value. Feature selection was performed using Random Forest (RF), Support Vector Machine (SVM), and Lasso algorithms to identify specific CpGs. The candidate CpGs were then tested in the remaining 30% of cases.

For external validation, we used datasets GSE90496, GSE161944, GSE50022, GSE64509, and EGAS00001004341, which included 121 H3K27M-mut gliomas, 24 IDH-mut gliomas, and 260 normal brain tissues. Primers were designed and validated using Mutation Capsule Plus (MCP), a technology enabling simultaneous detection of genetic and methylation alterations, as described in a previous study [33]. Ultimately, 76 primers were confirmed as the methylation detection panel.

### Library Preparation and sequencing analysis

DNA from CSF supernatant was isolated and quantified according to the protocols detailed in Supplementary Methods. Tissue samples underwent both DNA mutation and methylation testing. CSF samples with < 5 ng (Below the limit of detection, BLOD) were used for methylation library construction only, while those with ≥ 5 ng(Above the limit of detection, ALOD) were used for both mutation and methylation library construction.

MCP libraries were constructed from 5 ng of DNA following established protocols. ctDNA was first digested with methylation-sensitive restriction enzymes Hha I and Hinp 1I, then ligated with customized adapters and amplified to create the pre-MCP library. 400 ng of this pre-MCP library was digested with lambda exonuclease and amplified with gene-specific primers for target regions to construct the MCP library. Additionally, 1000 ng of the pre-MCP library was captured using a brain cancer panel of 68 genes (GenetronHealth, Beijing, China).

All libraries were sequenced on an Illumina NovaSeq 6000 platform following the manufacturer’s instructions. Sequencing reads were processed with a previously described bioinformatics pipeline [23, 33]. To evaluate the overall methylation signature of the 76 CpGs, we introduced the Methylation Signature Score (MSS) by following formula:

$$\begin{array}{l}\\\:Methylation\:Signature\:Score=\\\frac{\sum\:\left(H3\:specific\:markers\right)+(1-\sum\:\left(\:IDH\:specific\:makers\right))}{Probe\:Number}\end{array}$$

Details are provided in the Supplementary Methods.

### Development of methylation classifier model in tissue samples

To distinguish H3K27M-mut, IDH-mut, and Double-wildtype subtypes using methylation profiles, an RF classifier model was created to assign subgroups and corresponding probability using tissue methylation data of MCP. Model optimization was performed using tenfold cross-validation in the training cohort. Model performance was evaluated using the testing dataset based on sensitivity, specificity, and receiver operating characteristic (ROC).

Given the association of H3K27M with poor prognosis, we introduced the Methylation Risk Score (MRS), defined as:

$$\begin{array}{l}\\\:Methylation\:Risk\:Score=\\Predicted\:Probability\:of\:H3K27M\:in\:Methylation\:RF\:Models\end{array}$$

### Construction and evaluation of the co-detection classifier, termed BSGdiag

BSGdiag combines mutation and methylation data to determine glioma subtypes (Fig. 1B). For the H3K27M-mut subtype, if a mutation in H3F3A or HIST1H3B/C is present, the mutation-predicted probability is set to 1; otherwise, 0. For IDH-mut gliomas, an IDH1/2 mutation gives a mutation-predicted probability of 1. For the DW subtype, all mutation-predicted probabilities are 0. Methylation-predicted probabilities are obtained via the aforementioned methylation classifier model. The definitive diagnosis is ascertained based on the subtype with the highest probability.

For CSF data, we first assessed the diagnostic performance of the methylation classifier model on samples that completed methylation testing, evaluating sensitivity, specificity, AUC (Area Under the Curve), macro-AUC, and micro-AUC. Samples that underwent both mutation and methylation testing were utilized to assess the diagnostic performance of the BSGdiag classifier.

### Assessment of risk stratification of methylation features

The univariate Cox proportional hazards regression was employed to ascertain prognostic factors. Variables exhibiting a statistical significance of *P* < 0.05 were incorporated into a multivariate Cox regression analysis. A prognostic nomogram based on the multivariate Cox model was developed. Details are provided in the Supplementary Methods.

### Statistical analysis

For continuous variables consistent with normal distribution, the independent sample t-test and one-way ANOVA were used, while for continuous variables with non-normal distribution, the Mann-Whitney U-test and Kruskal-Wallis test were used. The Wilcoxon signed-rank test was used for paired samples in two groups, and the Friedman test was used for paired samples in multiple groups. For categorical variables, the chi-square test or Fisher exact probability method was used. Correlation analyses were performed using the Pearson correlation coefficient.

Sensitivity and specificity were calculated as true positive rate and true negative rate, respectively, with 95% confidence intervals estimated using the Agresti-Coull method. ROC curves and AUCs were computed, and their 95% confidence intervals were estimated using nonparametric bootstrapping (*R* = 100 iterations) with equi-tailed two-sided intervals. Survival analysis was performed using univariate and multivariate Cox hazard regression analysis.

Those P-values with two-tailed *P* < 0.05 were considered statistically significant. All graphs and statistical analyses were done using R software(v4.3.2) and detailed in Supplementary Methods.

## Results

### Patients, samples, and the study design

From October 2018 to July 2023, 80 patients with histopathologically confirmed brainstem gliomas were included. Clinical, MRI, pathological, and follow-up characteristics for patients with different subtypes are presented in Fig. 1C and summarized in Supplementary Table S1. Briefly, the cohort’s median age was 15 years (IQR, 8.8–33.2), with DIPG comprising 46.2% of cases. 51 patients (63.75%) were diagnosed with DMG, H3K27-altered, 7 patients (8.75%) with astrocytoma, IDH-mutant, and 22 patients (27.5%) with double-wildtype status, of which 54.5% (12 cases) were pilocytic astrocytoma (PA) and 27.3% (6 cases) were glioblastoma multiforme (GBM).

After MCP libraries and sequencing, methylation profiles were obtained from 138 CSF and 71 tissue samples, while mutation data were available for 69 CSFs and 71 tissues. In addition, 1016 tissue methylation profiles were extracted from public datasets used for the de novo biomarker design. Together, 1225 methylation sequencing profiles and 140 mutation sequencing profiles were employed for the whole study.

### Specific CpG sites for H3K27M and IDH mutations enabled reliable classification in multiple public cohorts

**Fig. 2:**
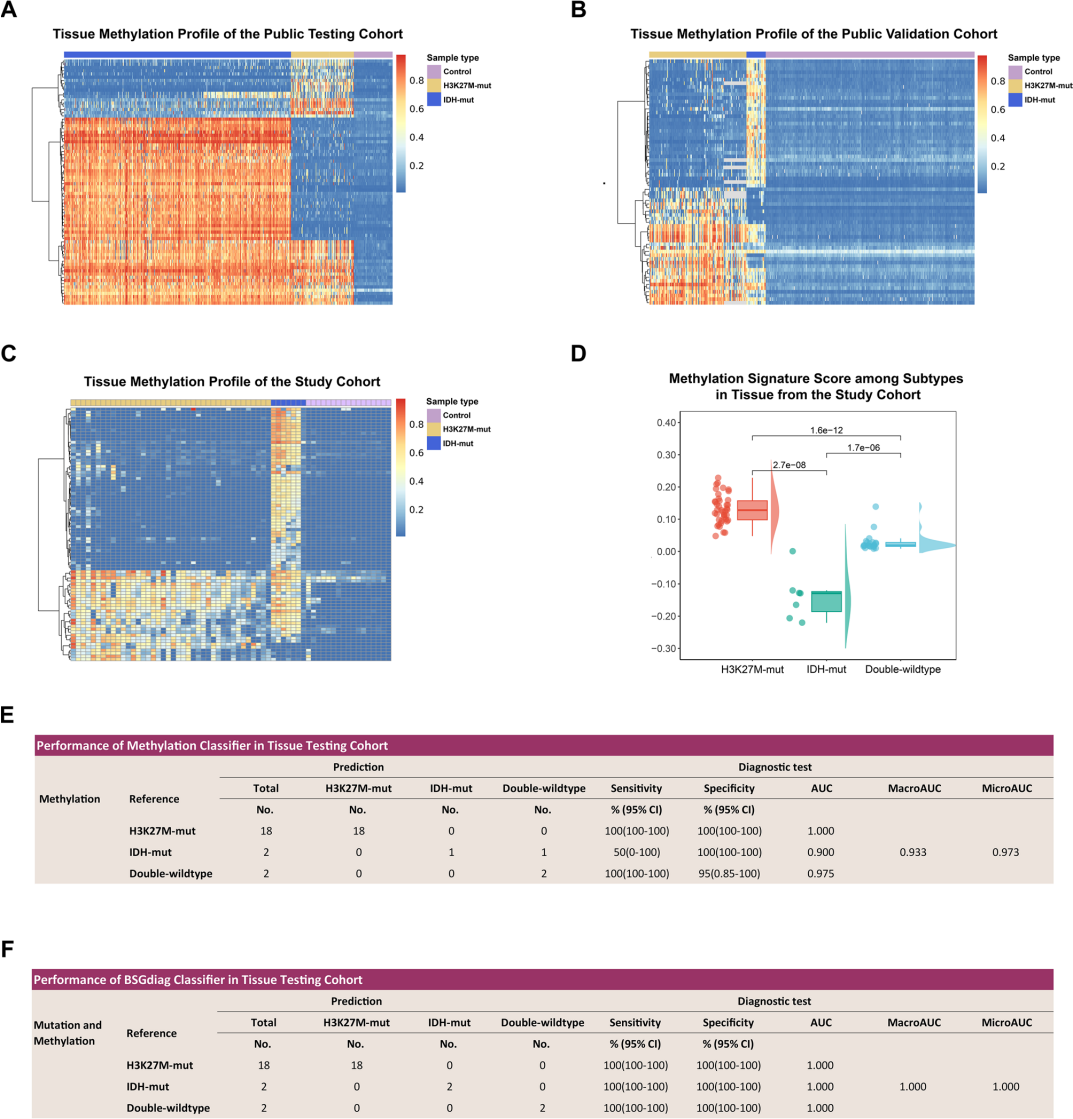
Subtype-specific methylation profiles and classification accuracy in public datasets and BSG tissue samples. (**A-C**) Heatmaps displaying the methylation ratios of highly differentially methylated CpG loci across H3K27M-mutant, IDH-mutant, and control samples. Each column represents an individual CSF sample, with the corresponding molecular subtype indicated at the top.Each row represents a single CpG locus. The color gradient represents the methylation ratio of each individual CpG site, ranging from 0 (unmethylated) to 1 (fully methylated). H3-specific markers refer to sites that are consistently hypermethylated in H3K27M-mutant cases, while IDH-specific markers are those that exhibit hypomethylation in H3K27M cases but hypermethylation in IDH-mutant cases. (**A**) Methylation profile of of 168 CpG sites in the testing cohort from public datasets. (**B**) Methylation profile of of 168 CpG sites in the validation cohort from public datasets. (**C**) Methylation profile of of 76 CpG sites in newly assayed tissue samples from the study cohort. (**D**) Cloud plot showing the distribution of the Methylation Signature Score across molecular subtypes in tissue, derived from 76-site methylation profiling. (**E**) Diagnostic performance of the methylation-based classifier in the tissue testing dataset. (**F**) Diagnostic performance of the mutation-methylation co-detection classifier (BSGdiag) in the tissue testing dataset Abbreviations: SNP, single nucleotide polymorphism; DEL, deletion; DNP, duplication; INS, insertion; A, astrocytoma; DMG, diffuse midline glioma; GBM, glioblastoma multiforme; LGG, low-grade glioma; PA, pilocytic astrocytoma; AUC, area under the curve

To identify a reduced set of differentially methylated CpG sites that could enable the classification of BSGs, highly differential CpGs were screened by reanalyzing previously published genome-wide DNA methylation array data (Supplementary Figure S1). Specifically, 24,068 highly discriminative differential CpG sites were found between DMG and IDH-mutant, 11,627 between DMG and controls, and 18,248 between IDH-mutant and controls. Then, by utilizing the algorithms for feature selection, a set of 168 CpGs was identified. As shown in the test cohort (Fig. 2A) and the external validation cohort (Fig. 2B), the 168 loci exhibited significantly unique subgroup characteristics.

Following the design, test and validation of primers, a panel of 76 CpGs primers were candidates for testing and external validation. The 76 CpGs sites achieved perfect AUC scores of 1.000, effectively distinguishing H3K27M-mutant, IDH-mutant, and control samples from each other in both testing and validation cohorts (Supplementary Figure S2). Similarly, the microAUC and macroAUC for the three-class classification also reached 1.000 across these cohorts. Differentially methylated CpG sites with gene annotation and subtype expression profiles derived from dataset EGAS00001004341 were shown in Supplementary Table S2.

### Integrated mutation and methylation profiling using targeted panels revealed distinct molecular features across BSG subtypes in tissue

Panels for gene mutations (68 genes) and DNA methylation (76 CpG sites) were utilized to co-detect mutations and methylation in 71 paired tumor tissues and blood samples. Genetic analysis revealed that H3K27M and IDH mutation were mutually exclusive (Supplementary Figure S3). Of 42 H3K27-mutant gliomas, 90.5% had H3F3A mutations, 7.1% were HIST1H3B-mutant, and 2.4% were negative for both H3F3A and HIST1H3B/C mutations. Within the H3K27M-mutant subtype, 76.2% had TP53 mutations and 52.3% had ATRX mutations. All mutations of FGFR1, PIK3CA, PPM1D, and most mutations of NF1 and PDGFRA were found in this subtype. All IDH-mutant gliomas had IDH1 mutations, often accompanied by TP53 and ATRX mutations. The DW subtype had fewer mutations, with all TERT and KRAS mutations exclusively found within it.

The heatmap in Fig. 2C illustrates the differential methylation patterns across H3K27M-mutant, IDH-mutant, and DW subtypes validated in the tissue analysis by the methylation panel (76 CpG sites). All H3K27M-mut gliomas, but one, showed high methylation ratio at H3K27M-specific sites and low at IDH-specific sites. Conversely, IDH-mutant gliomas demonstrated hypermethylation at all the 76 sites, while the DW subtype had low methylation across most genomic sites. For subtype-signature analysis (Fig. 2D), the Methylation Signature Scores, ranging from − 1 to 1, were highest in the H3K27M-mut group (median 0.128; IQR 0.098–0.157), compared to the DW (0.019; IQR 0.016–0.028) and IDH-mut groups (-0.129; IQR − 0.186 to -0.124). Significant differences were validated between each pair (*P* < 0.001). Furthermore, our analysis revealed a positive correlation between the variant allele frequency (VAF) of H3F3A mutations and MSS (*R* = 0.51, *P* = 0.001; Supplementary Figure S4) in tiusses, whereas an inverse correlation was discerned between IDH1 VAF and MSS (*R*=-0.89, *P* = 0.007).

### Methylation and co-detection classifiers achieved high diagnostic accuracy in tissue training and testing cohorts

We developed an RF classification model for methylation using a training set of 23 H3K27M-mut cases, 5 IDH-mut cases, and 17 DW cases, and optimized it through tenfold cross-validation. The three-class classifier demonstrated exceptional accuracy within the primary training set, with AUCs of 1.000 for the H3K27M-mut, IDH-mut, and DW subtypes versus the remaining classes (Supplementary Table S3).

Subsequently, we assessed the performance of this methylation classification model on the tissue testing set (Fig. 2E), comprising 18 H3K27-mut cases, 2 IDH-mut cases, and 2 DW cases. The AUC values were 1.000 for the H3K27M-mut subgroup, 0.900 for the IDH-mut subgroup, and 0.975 for the DW subgroup when evaluated against other classes. The macroAUC and microAUC were 0.933 and 0.973, respectively, indicating high classification accuracy.

Following this, we evaluated the diagnostic efficacy of the co-detection classifier, BSGdiag, in tissue samples (Fig. 2F). This model rectified the misclassification of one IDH-mutant patient identified as DW by the methylation model, achieving AUCs of 1.000 across all comparisons. This result underscores the applicability of BSGdiag in tissue-based diagnostics.

### Parallel mutational patterns in CSF liquid biopsies and corresponding tumor tissue

**Fig. 3:**
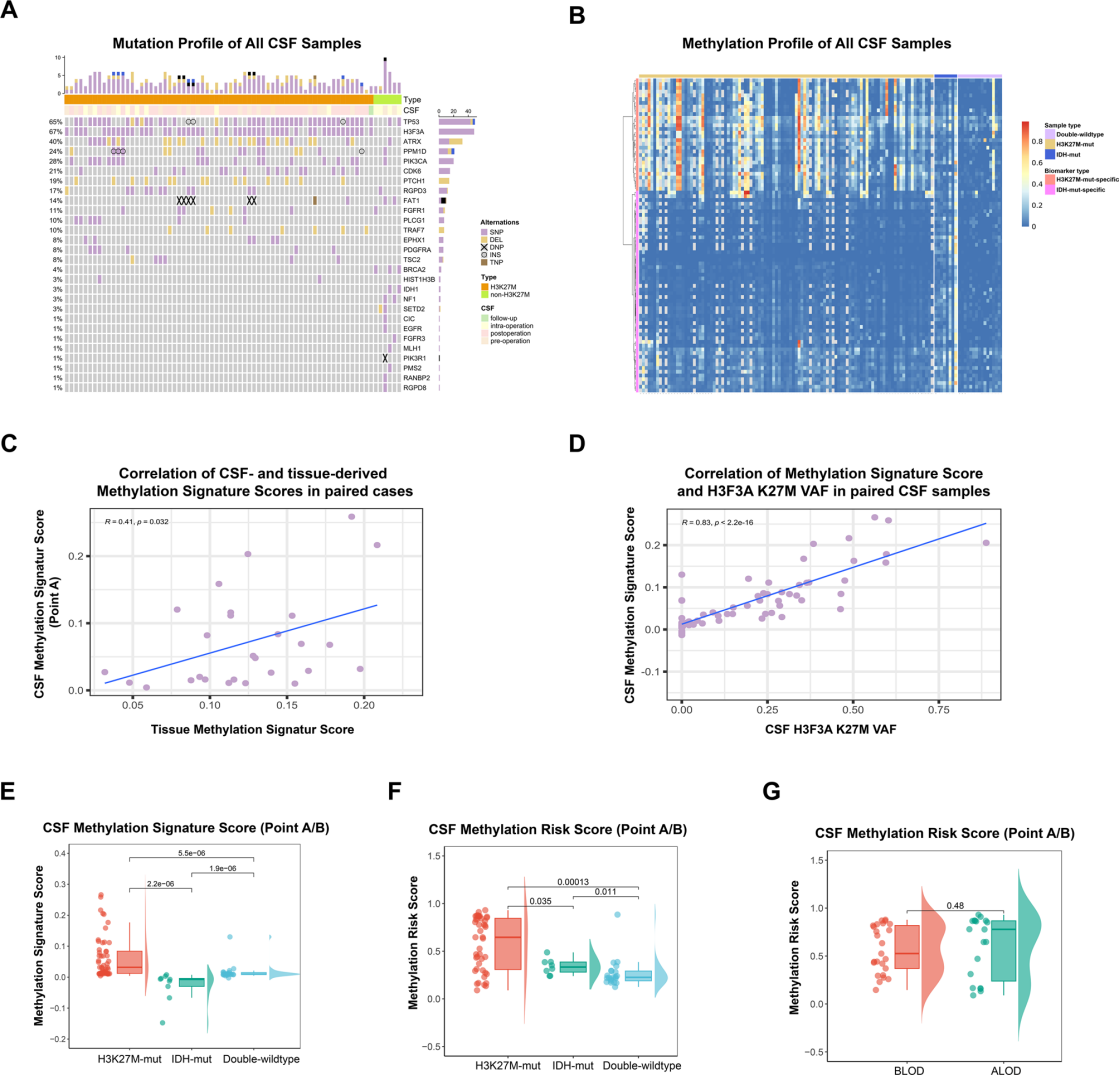
Cerebrospinal fluid (CSF) ctDNA mutation and methylation profiles. **A**. Landscape of genetic alterations detected in CSF-ctDNA. Each column represents an individual sample. The number of mutations per sample is shown at the top, followed by the molecular subtype and CSF source. The Genes displayed represent the top 10% most frequently mutated genes. The proportion of samples harboring mutations in each gene is indicated on the right. **B**. Heatmap displaying the methylation profile of 76 selected CpG loci across H3K27M-mutant, IDH-mutant, and double-wildtype CSF samples. Each column represents a single CSF sample, with molecular subtype annotated above. Each row represents a single CpG locus.The color gradient reflects the methylation ratio at each CpG site, ranging from 0 (unmethylated) to 1 (fully methylated). Gray dots represent missing values. **C**. Scatter plot showing the correlation between Methylation Signature Scores derived from preoperative CSF (Point A) and their paired tissue samples. The X-axis represents the tissue-derived Score and the Y-axis represents the CSF derived- Score. The concordance was calculated by the Pearson correlation coefficient. **D**. Scatter plot showing the correlation between CSF Methylation Signature Scores and H3F3A K27M VAFs in matched CSF samples. The X-axis indicates CSF H3F3A K27M VAF and the Y-axis indicates the CSF Methylation Signature Score. Pearson correlation was used to assess concordance. **E**. Raincloud plot illustrating the distribution of Methylation Signature Scores across molecular subtypes in CSF. **F**. Raincloud plot illustrating the distribution of Methylation Risk Scores across molecular subtypes in CSF. **G**. Raincloud plot comparing Methylation Risk Scores in pre-pre- and intra-operative CSF samples between BLOD and ALOD subgroups as determined by quantitative analysis of ctDNA Abbreviations: SNP, single nucleotide polymorphism; DEL, deletion; DNP, duplication; INS, insertion. CSF, cerebrospinal fluid. VAF, variant allele frequencie; BLOD, below the limit of detection; ALOD, above the limit of detection

We identified mutations in 71 CSF samples, with critical mutations and their frequencies displayed in Fig. 3A. Supplementary Figure S5A revealed a significant overlap between the CSF and tissue mutation profiles, with only 10 of the 68 examined gene mutations detected exclusively in tissues, while the remaining 85.3% of mutations were either present in both CSF and tissue samples or unique to CSFs.

High-frequency mutations such as H3F3A/TP53/PPM1D/IDH1 exhibit similar mutation rates between tissue and CSF samples, whereas low-frequency mutations like FGFR/NF1 are notably less frequent in CSFs (Supplementary Figure S5B). Despite a high positive rate of H3F3A mutations in preoperative or intraoperative CSF from H3F3A-positive tissues (95%, 19/20), the VAFs of H3F3A in CSF did not significantly correlate with tissue VAFs (*R* = 0.28, *P* = 0.35, Supplementary Figure S6). These findings suggest that a single CSF mutation analysis may not fully represent the tumor’s molecular alterations, due to tumor heterogeneity and the complexities of sample collections.

### CSF methylation levels exhibit significant discrimination among subtype

The DNA methylation characteristics of CSFs showed significant concordance with those of tissue samples. Methylation profiling was conducted on 134 samples, including 53 from timepoint A, 32 from timepoint B, 44 from timepoint C, and 5 from timepoint D. The heatmap of CSF methylation features was illustrated in Fig. 3B. We observed a moderate correlation (*R* = 0.41, *P* = 0.032) between ctDNA methylation Signature Scores in CSFs and their corresponding tissues (Fig. 3C). Additionally, mutation frequency and MSS were strongly correlated in CSFs (*R* = 0.83, *P* < 0.001, Fig. 3D).

Consistent with tissue samples, CSF Methylation Signature Scores varied significantly across three pathological subtypes (Fig. 3E), with all pairwise and overall statistical comparisons yielding P-values less than 0.001. Nevertheless, in DMG patients, we found that CSF methylation levels might be influenced by ctDNA content. Notably, the methylation MSSs of BLOD samples (0.0277, IQR 0.0122–0.0566) were lower compared to those of ALOD (0.0692, IQR 0.0124–0.159), with the difference nearly approaching statistical significance (Supplementary Figure S7, *P* = 0.055).

### RF model for methylation reduces ctDNA content impact on CSF methylome

The CSF Methylation Risk Scores (MRS, detailed in methods) were applied to assess the discriminative power and stability of the methylation RF model. Significant differences were observed among the subtypes (*P* < 0.05, Fig. 3F). The median methylation risk scores were 0.646 (IQR, 0.331–0.844) for the H3K27M-mut subtype, 0.334 (IQR, 0.281–0.386) for the IDH-mut subtype, and 0.223 (IQR, 0.190–0.292) for the DW subtype.

Moreover, compared to Methylation Signature Scores, RF model-derived Methylation Risk Scores demonstrated improved stability. The MRSs was 0.526 (IQR, 0.3370–0.818) in the BLOD group and 0.778 (IQR, 0.239–0.867) in the ALOD group, with no significant difference between the two groups (Fig. 3G, *P* = 0.48). This suggests that RF model-based classifiers may help mitigate the risk of diagnostic efficiency loss due to low ctDNA content, making them more suitable for clinical use.

### The co-detection classifier achieved optimal diagnostic performance in CSF

**Fig. 4:**
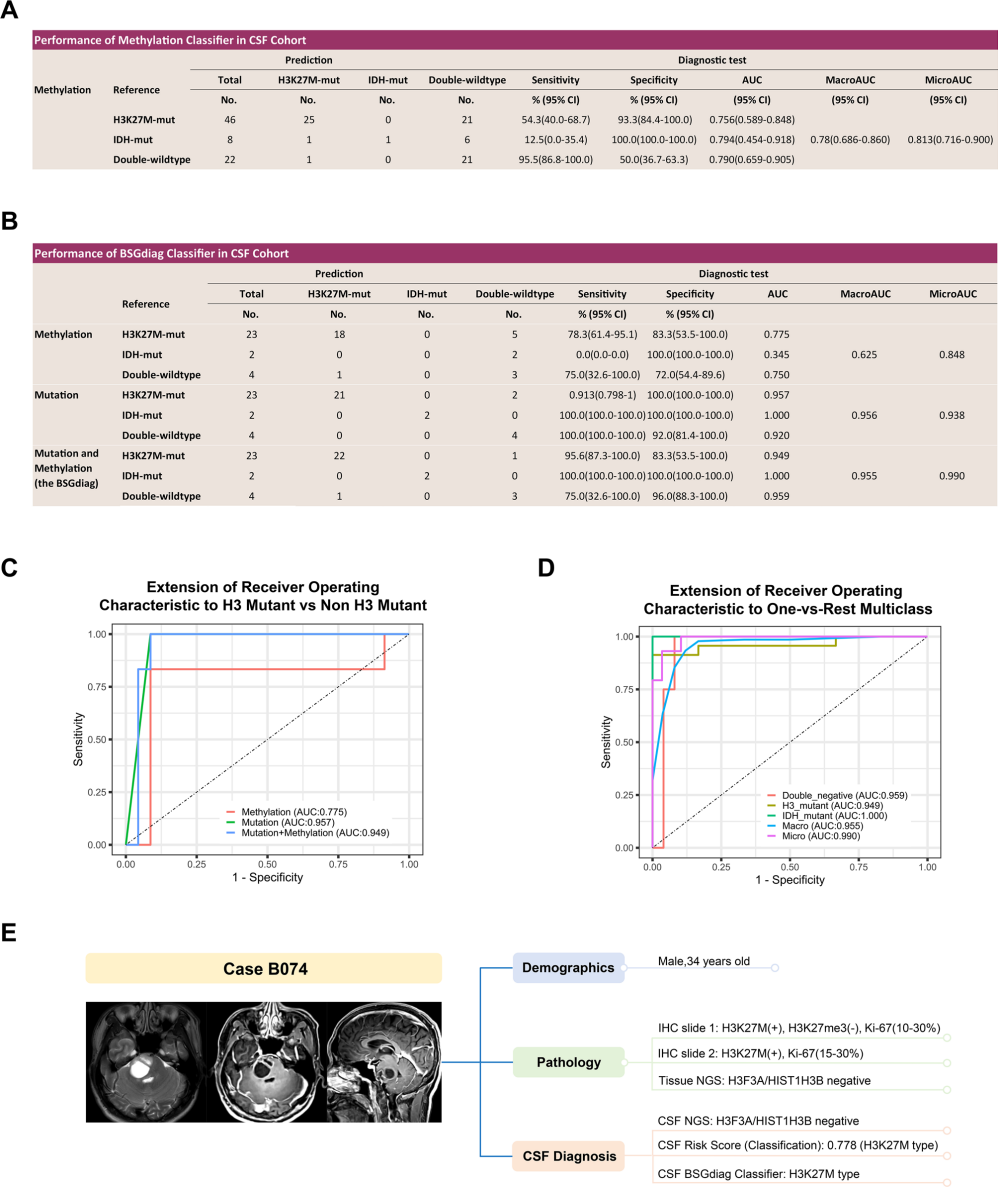
Diagnostic efficacy of the methylation model and mutation and methylation integrated diagnostic model (BSGdiag) in CSF Samples. **A**. Diagnostic performance of the methylation classifier in all pre- and intra-operative CSFs. Evaluation metrics include sensitivity, specificity, AUC, as well as macro- and micro-AUC values for multi-class classification. **B**. Diagnostic performance of the BSGdiag classifier in the pre- and intra-operative co-detection CSF samples. Performance metrics include sensitivity, specificity, AUC, as well as macro- and micro-AUC values for multi-class classification. Results based on methylation-only, mutation-only, and integrated detection are presented separately. **C**. ROC curves illustrating the performance of methylation-only, mutation-only, and combined BSGdiag models in distinguishing H3-mutant from non–H3-mutant tumors in CSF samples with co-detection. AUC values are indicated in the figure. **D**. One-vs-rest ROC curves of the BSGdiag classifier for multi-class subtype classification in pre- and intra-operative CSF samples with co-detection; corresponding AUC values are indicated. **E**. Representative case illustrating the application of BSGdiag in pre- and intra-operative CSF samples with co-detection. Shown are the patient’s preoperative MRI, clinical and pathological features, and CSF-based results from mutation-only analysis, the methylation-based RF model, and the integrated BSGdiag classifier, demonstrating its potential to assist in differential diagnosis when molecular findings are inconsistent with radiological and histopathology Abbreviations: CSF, cerebrospinal fluid; AUC, area under the curve; ROC, Receiver operating characteristic; RF, random forest

Based on the aforementioned results, we conducted a further assessment of the classifiers’ diagnostic performance in CSFs, including the methylation classifier and the BSGdiag. For the methylation classifier, all pre- and intra-operative CSF samples with ctDNA methylation detection were included (Fig. 4A). For the H3K27M-mutant subtype, the methylation classifier achieved a sensitivity of 54.3% (95% CI, 40.0- 68.7), specificity of 93.3% (95% CI, 84.4–100.0), and an AUC of 0.756. The AUCs for the IDH-mutant and DW subgroups were 0.794 and 0.790, respectively. The classifier yielded a macroAUC of 0.780 and a microAUC of 0.813, indicating acceptable three-class classification accuracy.

The BSGdiag achieved optimal diagnostic outcomes in CSF samples (Fig. 4B-D). For the H3K27M, the sensitivity reached 95.6% (95% CI, 87.3–100.0), and specificity was 83.3% (95% CI, 53.5 − 100.0%). AUCs were 0.949 for H3K27M-mut versus non-H3K27M-mut subtypes, 1.000 for IDH-mut, and 0.959 for DW. The macroAUC and microAUC reached 0.955 and 0.990, respectively (Fig. 4D).

Compared to mutation detection alone, the BSGdiag improved sensitivity for H3K27M-subtype (95.6% vs. 91.3%) but reduced specificity (83.3% vs. 100%), with the AUC for H3K27M detection remained nearly the same (0.949 vs. 0.957, Fig. 4B-C). This compromise makes sense considering the worst prognosis of H3K27M-mut BSG, particularly in the setting of liquid biopsy with limited ctDNA amount. For example, patient B074 (Fig. 4E), pathologically diagnosed as DMG, had negative H3K27M mutation tests in both tissue and CSF. However, a high methylation risk score (0.778) in CSF led to a final classification of H3K27M-mut glioma by BSGdiag, addressing false negatives in mutation testing. The AUC for IDH detection remained unchanged (1.000–1.000), while the AUC for DW improved (0.920 to 0.959). In terms of overall accuracy for three-class classification, the macroAUC remained stable (0.956 vs. 0.957), while the microAUC for three-class classification increased from 0.938 to 0.990.

### CSF Methylation Risk Score served as an independent risk factor for prognostic stratification

Next, we explored whether pre- and intra-operative CSF methylation features could offer a minimally-invasive approach for prognostic stratification, addressing the limitations of prognostic predictions based solely on mutation subtypes. Analyses were performed on 62 patients who completed methylation testing at timepoints A/B, including 36 with H3K27M-mut, 7 with IDH-mut, and 19 DW patients.

**Fig. 5:**
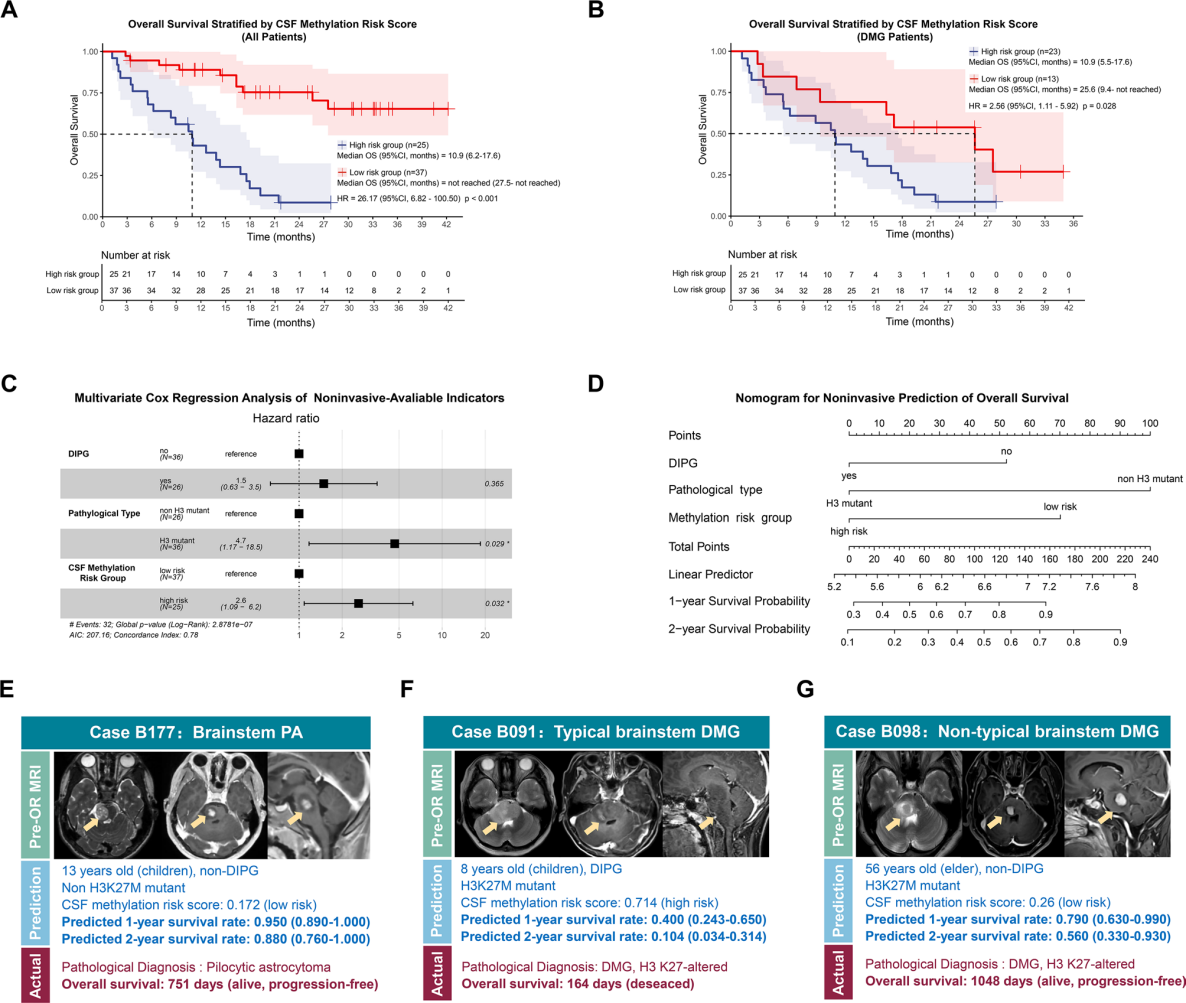
Stratification of patient prognosis by integrating of mutation analysis with CSF methylation profiling. (**A-B**). Kaplan–Meier curves illustrating overall survival in patients with pre- and intra-operative CSF samples, stratified by CSF Methylation Risk Score (cut-off value: 0.44). Median survival times with 95% CIs for each subgroup are shown on the right. HRs and p-values were calculated using Cox proportional hazards regression. (**A**) All patients; (**B**) Patients with DMG. **C**. Multivariate Cox proportional hazards analysis for all cases with pre- and intra-operative CSF samples, based on noninvasively available indicators, including DIPG diagnosis, H3 mutation status, and CSF Methylation Risk Group. Additional variables and their corresponding HRs and p-values from both univariate and multivariate analyses are provided in Supplementary Table S4. **D**. Nomogram of the multivariate Cox model developed from pre- and intra-operative CSF samples, representing prognostic prediction based on noninvasively available indicators. (**E**–**G**) Representative cases illustrating the consistency between predicted prognosis and actual outcomes.Yellow arrows indicate the lesions. (**E**) Brainstem pilocytic astrocytoma (PA); (**F**) Typical brainstem DMG; (**G**) Atypical brainstem DMG Abbreviations: CSF, cerebrospinal fluid; CI, confidence interval; HR, Hazard ratios; DMG, diffuse midline glioma; DIPG, diffuse intrinsic pontine glioma

The prognosis of BSG patients was stratified using the CSF Methylation Risk Score, employing a cutoff value of 0.44 to effectively differentiate high and low-risk groups (Fig. 5A). High-risk patients exhibited significantly poorer survival (HR = 26.17, 95%CI, 6.82–100.50, *P* < 0.001), with a mOS of 10.9 months (95% CI, 6.2–17.6 months), whereas the low-risk group did not reach 50% mortality (mOS, months: Not Reached; 95%CI, months: 27.5-Not Reached). Notably, this 0.44 cutoff also facilitated prognostic stratification within the H3K27M mutant subgroup (Fig. 5B). The high-risk subgroup (HR = 2.56, 95%CI, 1.11–5.92, *P* = 0.028) exhibited a markedly shorter mOS of 10.9 months (95% CI, 5.5–17.6 months) compared to 25.6 months (95% CI, 19.4-Not Reached) in the low-risk subgroup.

Univariate Cox regression analysis of presurgical available factors identified the CSF methylation risk group stratified by MRS (*P* < 0.001), pathological type (*P* < 0.001), and DIPG status (*P* < 0.001) as significant prognostic factors. Other factors, including sex, age group, midbrain, pontine and medullary involvement, infiltration, and enhancement, did not show statistical significance (Supplementary Table S4).The multivariate Cox analysis (Fig. 5C) identified H3 mutation (HR = 4.67, 95% CI: 1.17–18.5, *P* = 0.029) and the high-risk CSF methylation group (HR = 2.61, 95% CI: 1.09–6.25, *P* = 0.032) as independent adverse prognostic factors. A Concordance Index of 0.78 was achieved, and the Calibration curve was illustrated in Supplementary Figure S8.

The Sankey diagram (Supplementary Figure S9) illustrates the distribution and concordance of the H3 mutant status, CSF methylation risk group, and the corresponding histopathological diagnoses. The nomogram of the multivariate Cox model was illustrated (Fig. 5D), and three representative cases highlighted the role of CSF methylation risk group as a significant adjunct to radiological and mutation features: B177, a non-H3K27M patient with a low CSF methylation risk, had excellent survival, aligning with a PA diagnosis (Fig. 5E). Within the DMG patients, B091 with a high CSF methylation risk had poor survival, dying 164 days after diagnosis(Fig. 5F), while B098 with a low CSF score had a favorable prognosis, surviving 1048 days after surgery (Fig. 5G).

### The temporal dynamics of molecular features in CSF elucidate the tumor burden

**Fig. 6:**
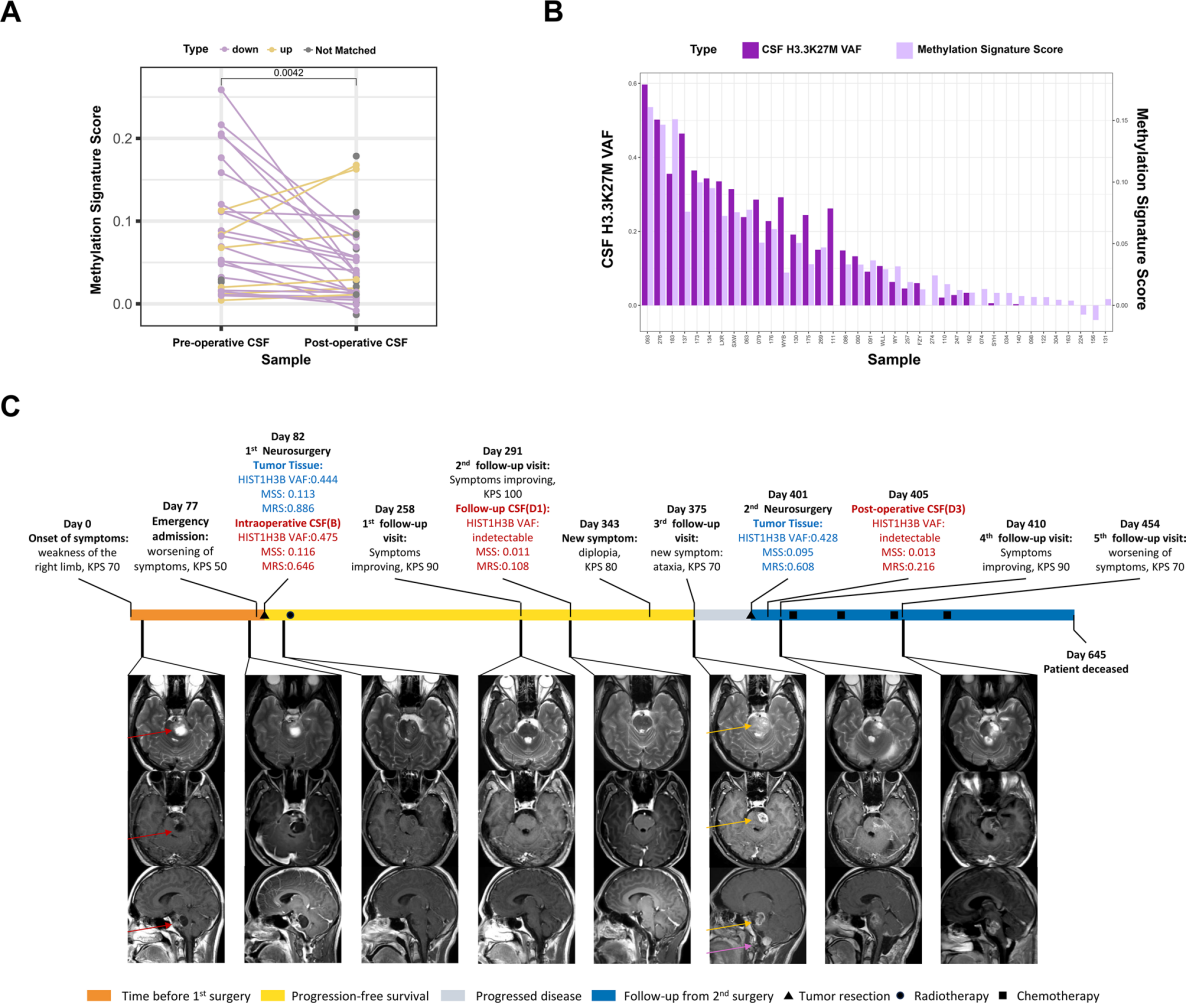
Co-detection of mutations and methylation in postoperative and follow-up samples. (**A**) Distribution of Methylation Signature Scores (MSS) in pre-operative and post-operative CSF samples, along with paired-sample comparisons. Purple dots and connecting lines indicate patients with a postoperative decrease in MSS relative to baseline; yellow dots represent increases; grey dots denote unmatched samples. (**B**) Paired bar plots comparing H3F3A variant allele frequency (VAF) and methylation levels in matched postoperative CSF samples with mutation/methylation co-detection. (**C**) Representative case (LGN) illustrating dynamic monitoring of CSF ctDNA in the patient with DMG. Colored bars represent sequential clinical phases. Eight sets of MRI below the demonstrate the dynamic changes in primary lesions (red arrows), recurrent lesions (yellow arrows), and disseminated lesions (purple arrows) from the first preoperative assessment to the final follow-up. Annotations above the timeline records changes in clinical status and the mutation/methylation co-detection results from tissue and/or CSF samples at corresponding timepoints Abbreviations: CSF, cerebrospinal fluid;. MSS, Methylation Signature Score; VAF, variant allele frequency; MRS, Methylation Risk Score

Postoperative Methylation Signature Scores significantly decreased compared to preoperative MSSs (*P* = 0.004, Fig. 6A), corresponding with reduced tumor burden. However, the Methylation Risk Score remained stable between the two timepoints (*P* = 0.260), demonstrating consistent diagnostic accuracy for H3K27M mutations irrespective of tumor burden (Supplementary Figure S10). Further analysis revealed no significant correlation between the decrease in MSS and tumor volume (*R* = 0.12, *P* = 0.46, Supplementary Figure S11). Moreover, among the 10 patients with undetectable H3F3A-mut VAF postoperatively, 8 (80%) cases exhibited detectable MSSs compared to the baseline (Fig. 6B).

We further assessed the dynamic variations in mutation and methylation features in follow-up CSFs from three patients. For an 18-year-old male with recurrent DMG (Fig. 6C), initial CSF showed a HIST1H3B-mut VAF of 0.475 and a methylation level of 0.11, reflecting the disease severity. After complete tumor resection and radiotherapy, the VAF dropped to undetectable levels by day 291, consistent with a complete clinical response assessed by MRI (Fig. 6C) and symptoms (Supplementary Figure S12) according to iRANO criteria (Fig. 6C). However, a residual MSS of 0.011 persisted in CSF at day 291, preceding symptom worsening by 52 days. Despite a second surgery with undetectable VAF, the MSS slightly elevated to 0.013 in postoperative CSF at day 405, indicating an MRD status post-second surgery. This persistent methylation signal accurately reflected ongoing disease activity. Ultimately, the disease progressed, leading to the patient’s death on day 645.

Two additional cases demonstrate congruency between CSF methylation fluctuations and tumor progression. In Supplementary Figure S13, the 12-year-old female exhibited a slight increase in CSF MSS post-surgery (from 0.068 to 0.084) and a further escalation post-radiotherapy (to 0.125), corresponding with rapid tumor dissemination from the brainstem to the lateral ventricles. Conversely, an 8-year-old male (Supplementary Figure S14) showed a significant decrease in MSS post-surgery (from 0.015 to 0.001), which then rose upon tumor recurrence, as measured by MRI on day 120 (to 0.039). These cases underscore the relationship between methylation dynamics and tumor progression.

## Discussion

BSG represents a group of challenging brain tumors with remarkable heterogeneity beyond the DMG, H3K27-alt framework by WHO CNS5^1,3,4^. Comprehensive genetic and epigenetic analysis is essential for effective treatment strategies [6, 20, 21, 34–38]. Despite the high risks of surgery or biopsy in the brainstem, BSG is ideal for CSF-based liquid biopsy due to its CSF immersion [23]. Building on our previous work with CSF ctDNA mutation sequencing and the potential value of non-mutational ctDNA fragments for methylation analysis [11, 23, 27]we developed and validated a BSG-specific methylation assay, leading to the creation of BSGdiag, a diagnostic model that combines mutation and methylation detection from CSFs, offering improved diagnostic accuracy. Additionally, CSF ctDNA methylation demonstrated superior sensitivity in prognosis and MRD monitoring. This study paves the way for future clinical applications and trial designs.

The brainstem, while part of the midline structures, is uniquely differentiated from the thalamus, cerebellum, and spinal cord, both in terms of surgical risks and molecular background. The use of DMG, H3K27-alt as a representation for brainstem gliomas is neither exhaustive nor specific. Nonetheless, the clinical urgency for a BSG-specific molecular classification is undeniable. The first innovative theoretical proposition of this study is the introduction of our unique molecular diagnostic framework for BSG, specifically H3K27-mut, IDH-mut, and DW subtypes.

Although this classification is more comprehensive and specific than DMG, H3K27-alt, it is not flawless. Driver mutations alone are insufficient; concurrent mutations also significantly influence prognosis [7, 39]. For instance, an isolated H3K27M mutation can occur in pilocytic astrocytomas [40]gangliogliomas [41]or ependymomas [42]. Tumors co-mutated with H3K27M-BRAF/FGFR1 tend to have a more favorable prognosis than those with H3K27M-TP53/PPM1D^7,41^. Therefore, our 68-gene panel exome sequencing provides a more comprehensive mutation profile, guiding effective treatment strategies.

Furthermore, epigenetic mechanisms, including DNA methylation, play an equally critical role as gene mutations in BSG [12, 43]. For instance, while the presence of an H3K27 mutation significantly influences BSG, the methylation profile can further stratify them into H3-Pons and H3-medulla methylation classes [9]. Additionally, different isoforms of the H3K27M mutation (H3F3A and HIST1H3B/C) have distinct methylation profiles and prognosis. Hence, the second innovative theoretical proposition of this study is the integration of DNA mutation and methylation data.

The primary technical advancement reported here is the successful realization of these two innovative theoretical propositions in the context of liquid biopsy for the first time. To address the scarcity of CSF and its low ctDNA abundance, which often hampers genome-wide methylation profiling using 450/850K arrays [16, 26, 27]we developed a methodology involving data mining and validation from public methylation databases, followed by tissue validation, model construction, and CSF validation. This process identified a stable 76-panel methylation signature across multiple sample types. While CSF diagnostic efficacy is lower than tissue, expanding the CSF sample size by adding one or two lumbar punctures could enhance model performance. This technique paves the way for exploring other methylation biomarkers in CSF, such as those predictive of drug/immunotherapy responsiveness [44].

BSGdiag achieved excellent diagnostic performance in CSF samples, with a three-class macro-AUC of 0.955 and a micro-AUC of 0.990. The sensitivity, specificity, and AUC for the H3K27M-mut subtypes were 95.6%, 83.3%, and 0.949, respectively, comparable to ddPCR-based studies [24, 45–47]. However, BSGdiag is suitable for diagnosis, prognosis, and disease monitoring, whereas ddPCR’s application is limited to known driver mutations, requiring prior tissue sequencing for comprehensive molecular information. Compared to mutation-based diagnosis, BSGdiag showed improved sensitivity but reduced specificity for the H3K27M subtype, and vice versa for the DW subtype (Fig. 4B). This is acceptable, considering the lower concentration of ctDNA in liquid biopsy, as missing a diagnosis in DW patients is less severe than in H3K27M patients.

Ideally, liquid biopsy should facilitate immediate commencement of tailored treatment, independent of tissue information. Recently, Cecilia Arthur administered MEK and BRAF inhibitors to a patient with inoperable brainstem tumors based on CSF detection of BRAF V600E mutation, resulting in substantial tumor regression and remarkable clinical improvement [48]. In the absence of tissue information, the advantages of BSGdiag are more pronounced. For example, if tissue were unavailable, patient B074 would not be diagnosed as DMG based solely on CSF ctDNA mutation tests. This may be due to VAF below the detection limit, or other allele mutations in histone H3 or EZHIP overexpression. A similar situation could arise in IDH-mutant tumors, where, in rare cases, the “IDH methylation class” may include those without IDH mutation [9]. DNA methylation can address these scenarios, reducing false negatives and improving sensitivity.

Due to its lack of specific CpG sites, the DW subtype is susceptible to misclassification by DNA methylation, especially when ctDNA is low. However, most DW patients belong to PA, which is predominantly found in the midbrain and medulla oblongata, making surgery or stereotactic biopsy riskier than in the pons [38]. Therefore, adding one or two lumbar punctures or collecting CSF during an endoscopic third ventriculostomy to acquire enough ctDNA is more acceptable than surgery. Additionally, an Ommaya reservoir is another optimal alternative for serial CSF collection [44].

CSF Methylation Rrisk Scores have been demonstrated as an independent prognostic factor, enabling both overall cohort and subgroup stratification within DMG cases. This aligns with our earlier tissue methylome research, which classified H3K27M patients into H3-Pons and H3-Medulla methylation subgroups with distinct prognoses [9]indicating that these 76 methylation markers may contain information on the cell of origin or tumor microenvironment. For disease monitoring, aligned with previous reports for plasm/CSF H3K27M VAFs in ONC201 treatment response [25]mutation VAF does not correlate linearly with tumor burden, but its trend can reflect tumor flunctutions in our cohort. CSF ctDNA methylation signal is potentially more sensitive than VAF and thus better suited for MRD detection and monitoring, as shown in the illustrated cases (Fig. 6C-E). Therefore, BSGdiag offers powerful tools for novel therapies, delivering timely, objective, comprehensive, and necessary information.

This study has several limitations. First, while our methylation markers and diagnostic model performed well in tissue-based analyses, the absence of DW subtype–specific methylation markers, particularly for pilocytic astrocytomas, may compromise the diagnostic accuracy of the CSF-based methylation classification—especially considering the limited abundance of ctDNA and the complexity of DNA fragment release in CSF. Further genome-wide CSF methylome profiling for marker discovery and validation, as reported in brain tumor studies (e.g., Zuccato et al., *Neuro Oncol*., 2023^11^), may help enhance diagnostic performance in this context.

Second, the relatively small and imbalanced sample size, especially for IDH-mut and DW subtypes, reflects both the rarity of brainstem gliomas and the predominance of the H3K27M subtype in our cohort. Given the limited scale of CSF-based DMG/BSG studies even in multicenter settings (e.g., Cantor et al., *Neuro Oncol.*, 2022: 29 CSF samples, mutation only [25]; Bruzek et al., *Clin Cancer Res*, 2020: 16 samples, mutation only [49]; Pan et al., *Acta Neuropathol.*, 2019: 57 samples, mutation only [23]; Panditharatna et al., *Clin Cancer Res*, 2018: 30 samples, mutation only [24]), these challenges highlight the urgent need for broad, multi-institutional collaborations to expand sample sizes and enhance generalizability.

Third, given the retrospective nature of our cohort, there is a possibility of biases in sample selection, clinical management, and follow-up duration, which cannot be fully excluded. Future larger, prospective and multicenter clinical studies with standardized sampling and longitudinal follow-up are warranted to further reduce bias, strengthen the level of evidence, and thus facilitate the clinical translation.

## Conclusion

In conclusion, this study focused on various BSG types, adopting a 2-driver gene-based molecular diagnosis as the gold standard and integrating DNA methylation for comprehensive analysis, to advance CSF ctDNA-based liquid biopsy of BSG. BSGdiag excelled in comprehensiveness, reliability, robustness, and minimal invasiveness, making it ideal for the holocyclic management of BSG. This study paves the way for future clinical applications and trial designs.

## Electronic supplementary material

Below is the link to the electronic supplementary material.

Supplementary Material 1

Supplementary Material 2

Supplementary Material 3

## Data Availability

All data generated in this manuscript are publicly available, access requires a request to the corresponding authors in accordance with institutional policies. Data was deposited in the China National Center for Bioinformation (CNCB) and shared at https://ngdc.cncb.ac.cn/gsa-human/s/nu499ZOa.

## Abbreviations

BSG: Brainstem glioma
CSF: Cerebrospinal fluid
ctDNA: Circulating tumor DNA
DIPG: Diffuse intrinsic pontine gliomas
DMG: Diffuse midline glioma
NCCN: National Comprehensive Cancer Network
EANO: European Association of Neuro-Oncology
ddPCR: Digital droplet polymerase chain reaction
NBTRC: National Brain Tumor Registry Center of China
DW: Double-Wildtype
OS: Overall survival
RF: Random Forest
SVM: Support Vector Machine
MCP: Mutation Capsule Plus
BLOD: Below the limit of detection
ALOD: Above the limit of detection
MSS: Methylation Signature Score
ROC: Receiver operating characteristic
MRS: Methylation Risk Score
AUC: Area Under the Curve
PA: Pilocytic astrocytoma
GBM: Glioblastoma multiforme
IQR: Interquartile range
HR: Hazard Ratio
