# Supplementary material for: Co-detection of mutations and methylations in cerebrospinal fluid ctDNA for minimally-invasive diagnosis of brainstem glioma
Source: J Exp Clin Cancer Res. 2025 Oct 7;44:283. doi: 10.1186/s13046-025-03455-y (PMC12502398; doi:10.1186/s13046-025-03455-y)
Supplement: Supplementary file 1 — Supplementary Material 1 [file 13046_2025_3455_MOESM1_ESM.docx]

**Supplementary Figures**

**Supplementary Figure S1.** Paradigm for screening, testing, and validation of H3K27M-mut and IDH-mut specific methylation probes based on public datasets.

**Supplementary Figure S2.** One-vs-rest of the ROC curves for 76 CpGs panel for multi-class classification in the public testing cohort (A) and validation cohort (B).

**Supplementary Figure S3.** Landscape of genetic alterations detected in Tissue.

**Supplementary Figure S4.** Scatter plot showing the correlation of tissue Methylation Signature Score and H3F3A/IDH mutation VAF.

**Supplementary Figure S5.** Comparison of Mutation Profiles Between CSF and Tumor Tissue.

**Supplementary Figure S6.** Scatter plot showing the correlation of tissue H3F3A mutant VAF and preoperative H3F3Amutation VAF.

**Supplementary Figure S7.** Raincloud plot comparing Methylation Risk Score in pre-pre- and intra-operative CSF samples between BLOD and ALOD subgroups as determined by quantitative analysis of ctDNA.

**Supplementary Figure S8.** Calibration curve to compare the predicted nomogram.

**Supplementary Figure S9.** Sankey diagram illustrating the relationships between patients' pathological type, CSF methylation group, and histopathological diagnosis.

**Supplementary Figure S10.** Distribution of Methylation Risk Scores (MRS) in pre-operative and post-operative CSF samples, along with paired-sample comparisons.

**Supplementary Figure S11.** Scatter plot showing the correlation of Methylation Signature Score reduction and tumor volume reduction (From pre-operation to post-operation).

**Supplementary Figure S12.** Photographs illustrating the clinical course of Case LGN from preoperative status to the second postoperative follow-up.

**Supplementary Figure S13.** Representative case (B137) illustrating dynamic monitoring of CSF ctDNA in patients with DMG. Colored bars represent sequential clinical phases.

**Supplementary Figure S14.** Representative case (ZJY) illustrating dynamic monitoring of CSF ctDNA in patients with DMG. Colored bars represent sequential clinical phases.


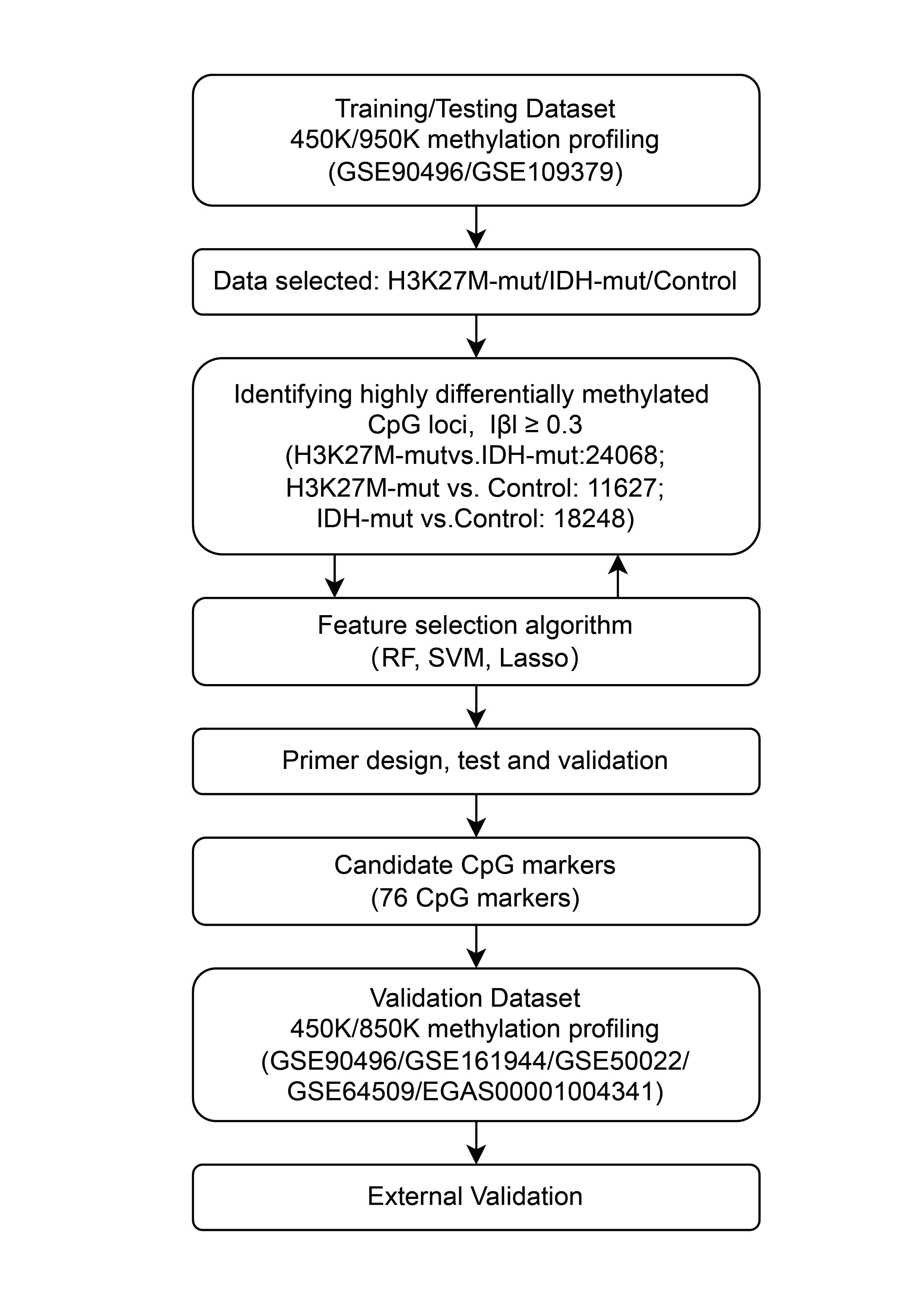


**Supplementary Figure S1.** Paradigm for screening, testing, and validation of H3K27M-mut and IDH-mut specific methylation probes based on public datasets.

**Abbreviations:** RF, Random Forest; SVM, Support Vector Machine.


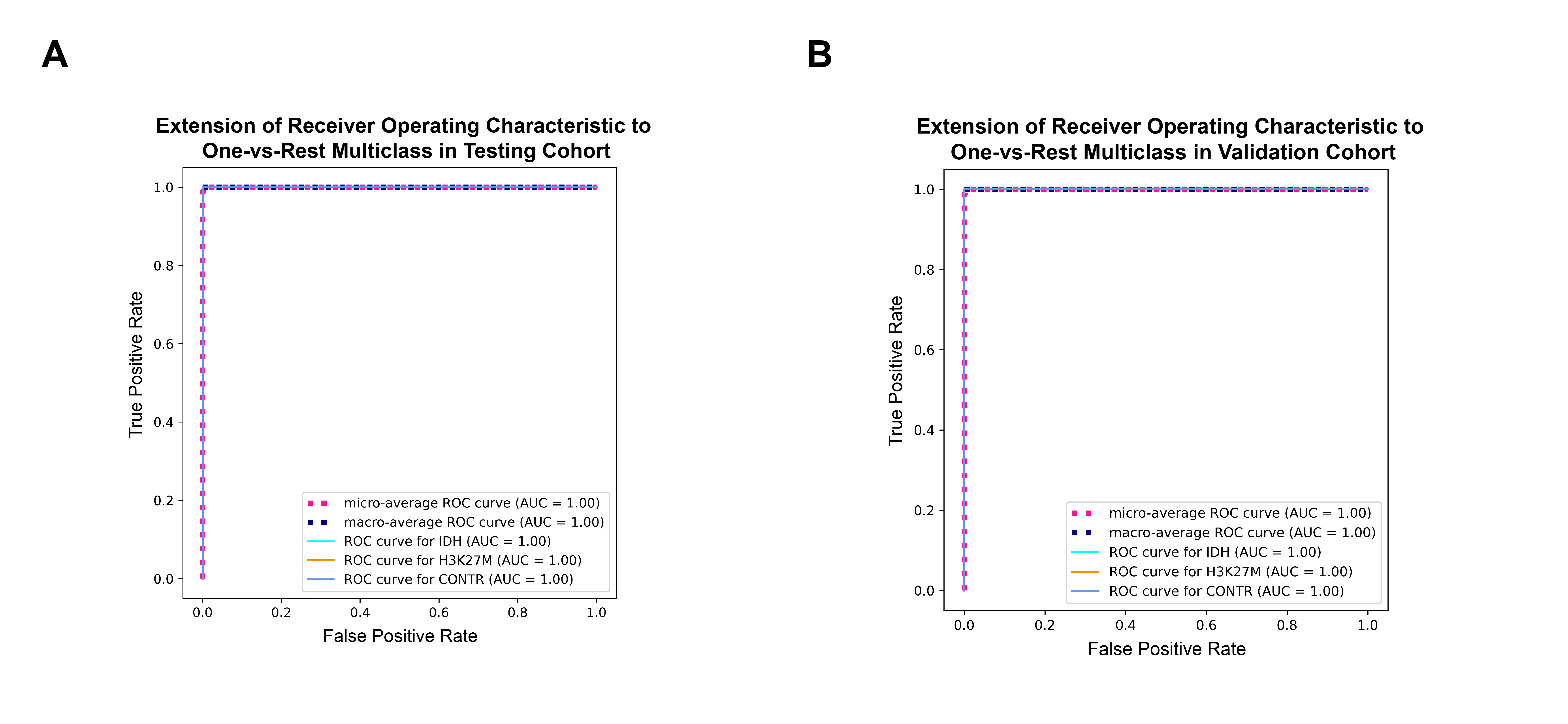
**Supplementary Figure S2.** One-vs-rest of the ROC curves for 76 CpGs panel for multi-class classification in the public testing cohort (A) and validation cohort (B). AUC values are indicated in the figure.

**Abbreviations:** ROC, Receiver operating characteristic.


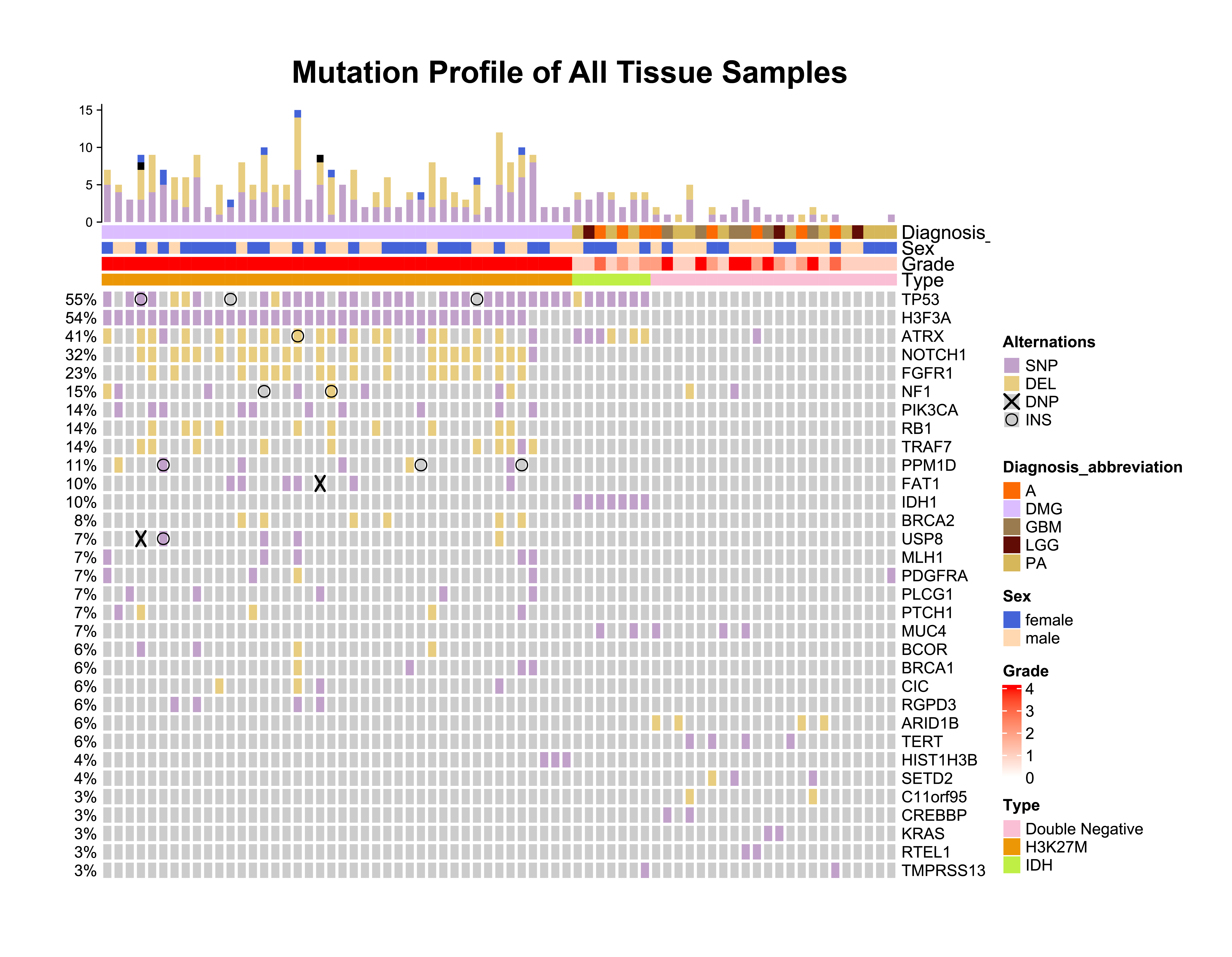


**Supplementary Figure S3.** Landscape of genetic alterations detected in Tissue. Each column represents an individual sample. The number of mutations per sample is shown at the top, followed by the molecular subtype and CSF source. The genes displayed represent the top 10% most frequently mutated genes. The proportion of samples harboring mutations in each gene is indicated on the right.

**Abbreviations:** CSF, Cerebrospinal Fluid.

**
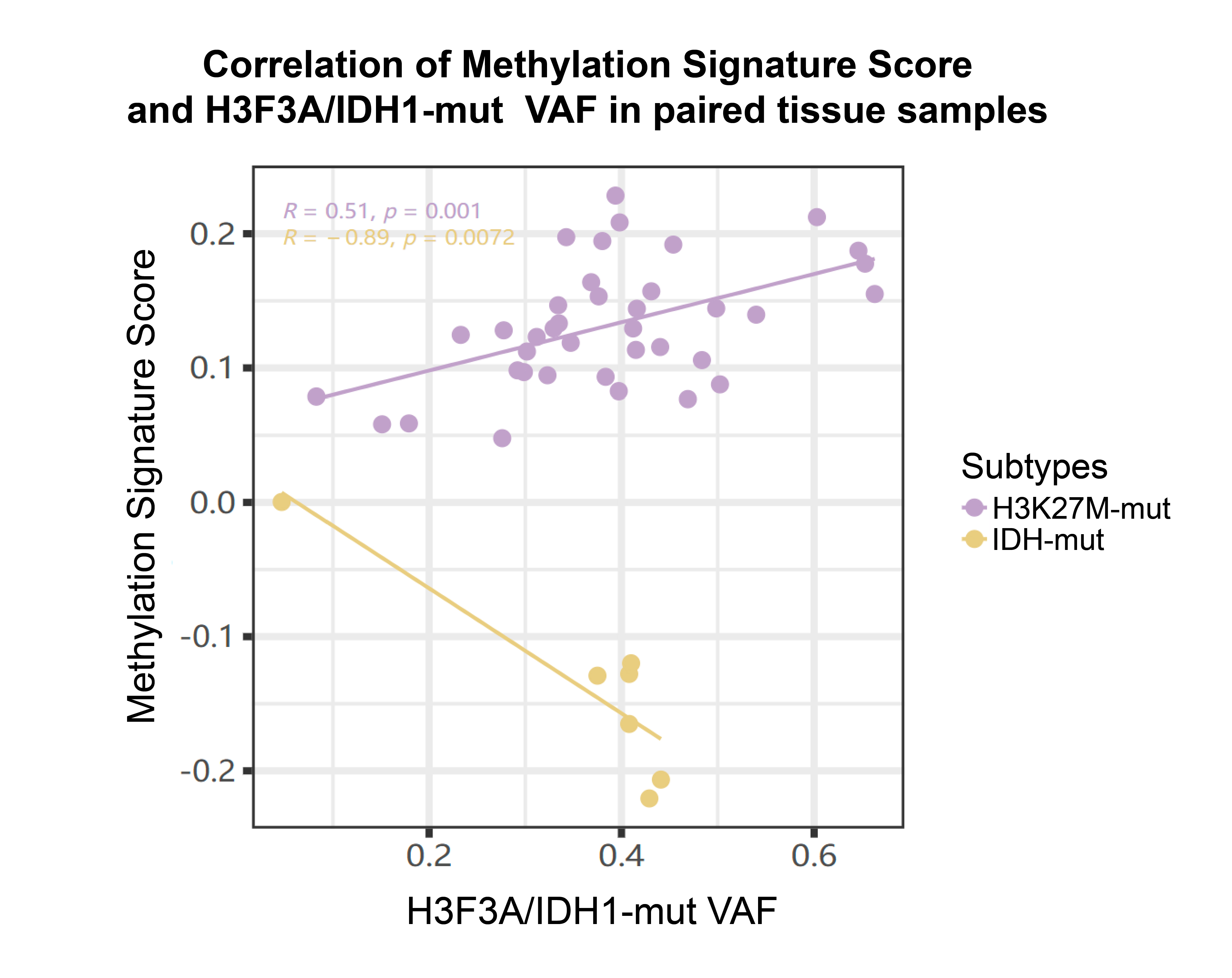
**

**Supplementary Figure S4.** Scatter plot showing the correlation of tissue Methylation Signature Score and H3F3A/IDH mutation VAF. The X-axis indicates CSF H3F3A/IDH mutation VAF and the Y-axis indicates the CSF Methylation Signature Score. Pearson correlation was used to assess concordance.

**Abbreviations:** VAF, Variant allele frequency; CSF, Cerebrospinal Fluid.

**
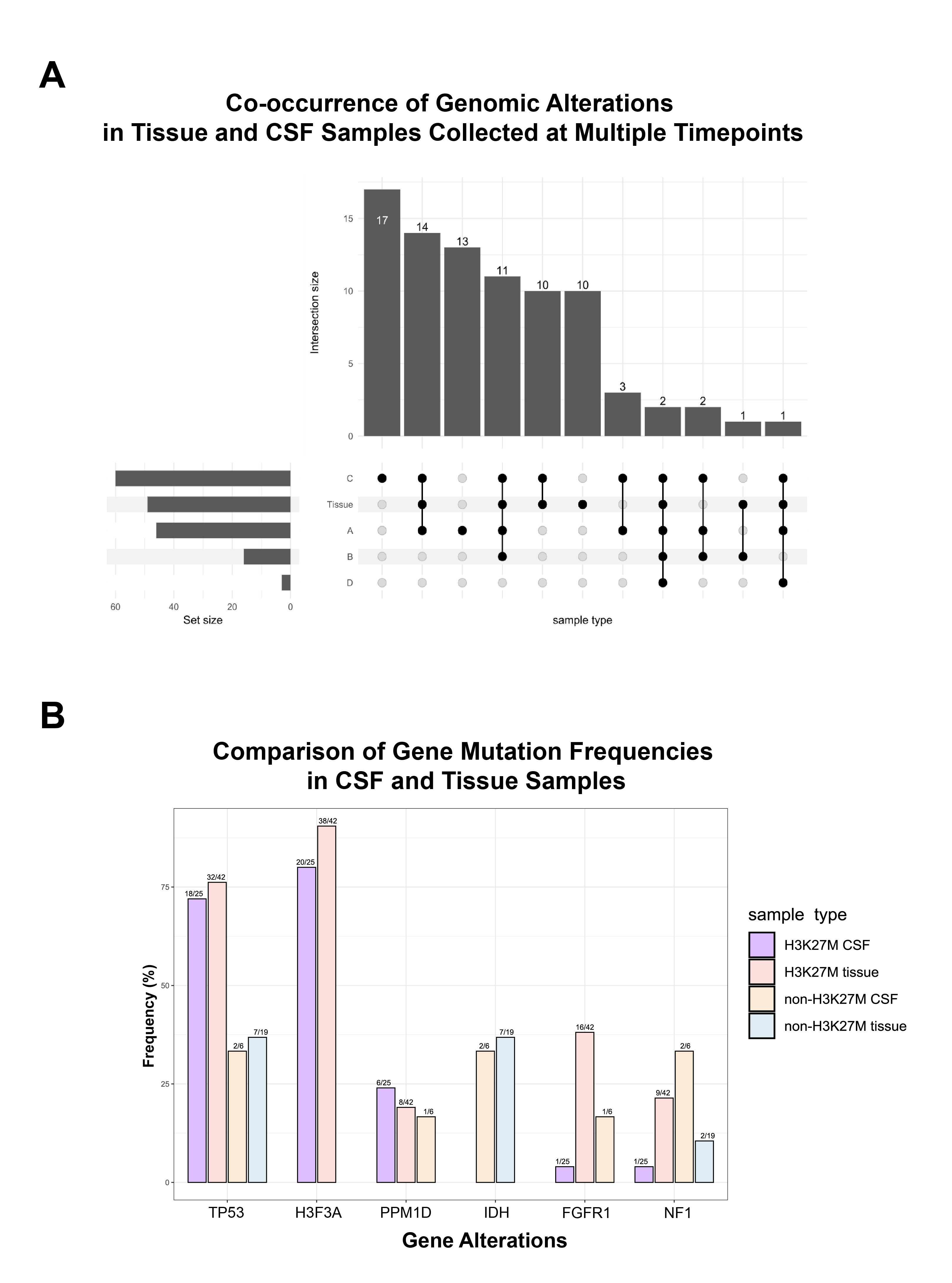
**

**Supplementary Figure S5.** Comparison of Mutation Profiles Between CSF and Tumor Tissue:

(A) UpSet plot showing the number of shared genomic alterations between tumor tissue and CSF samples collected at multiple timepoints.

(B) Comparison of key gene mutation frequencies—TP53, PPM1D, FGFR1, IDH, NF1, and F3F3A—in CSF and tissue samples. For each gene, mutation frequencies are individually displayed across H3K27M tissue, H3K27M CSF, non-H3K27M tissue, and non-H3K27M CSF groups.

**Abbreviations:** CSF, Cerebrospinal Fluid;

**
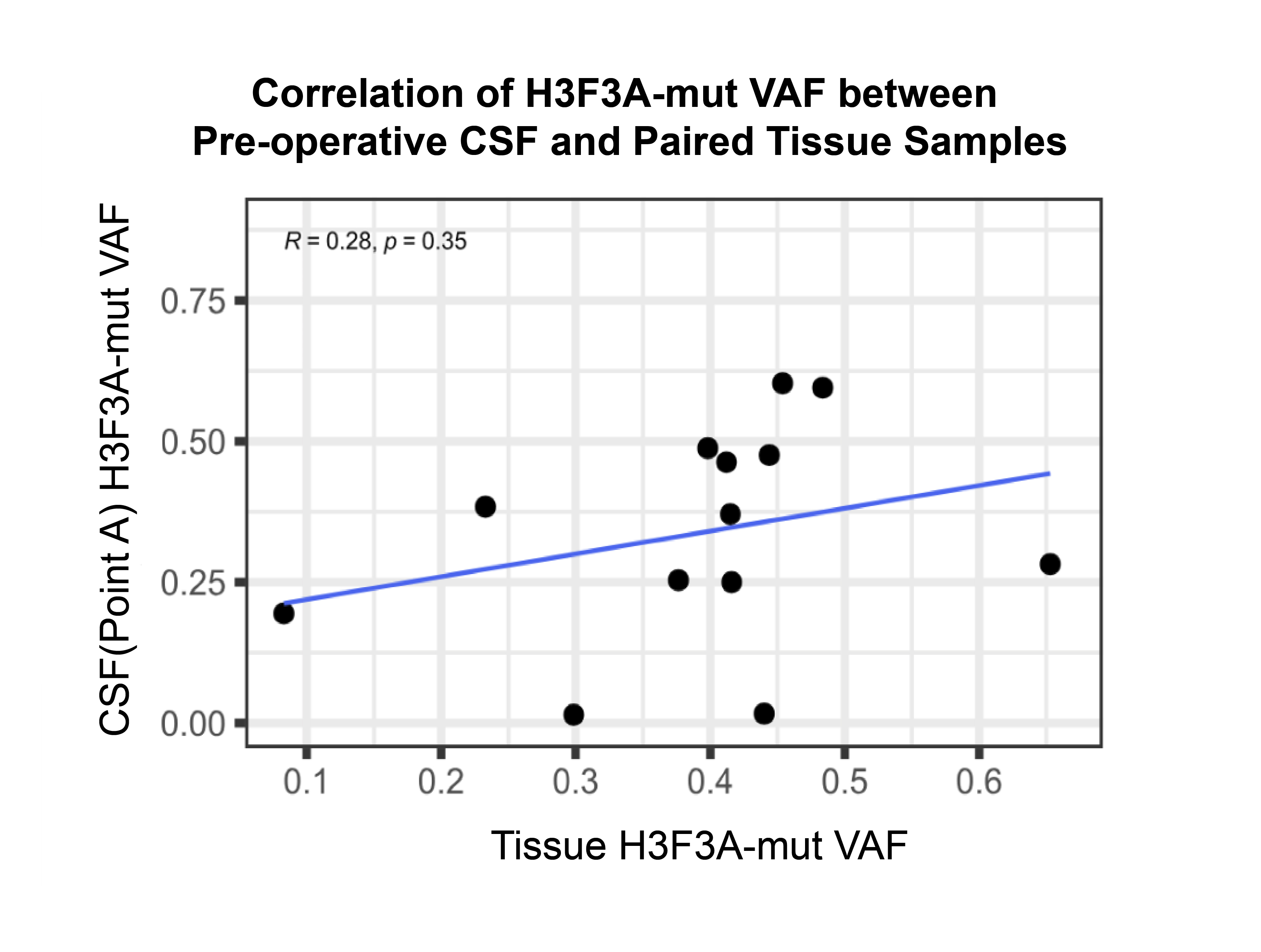
**

**Supplementary Figure S6.** Scatter plot showing the correlation of tissue H3F3A mutant VAF and preoperative H3F3Amutation VAF. The X-axis indicates tissue CSF H3F3A mutation VAF and the Y-axis indicates the preoperative CSF H3F3A mutation VAF. Pearson correlation was used to assess concordance.

**Abbreviations:** VAF, Variant allele frequency; CSF, Cerebrospinal Fluid.


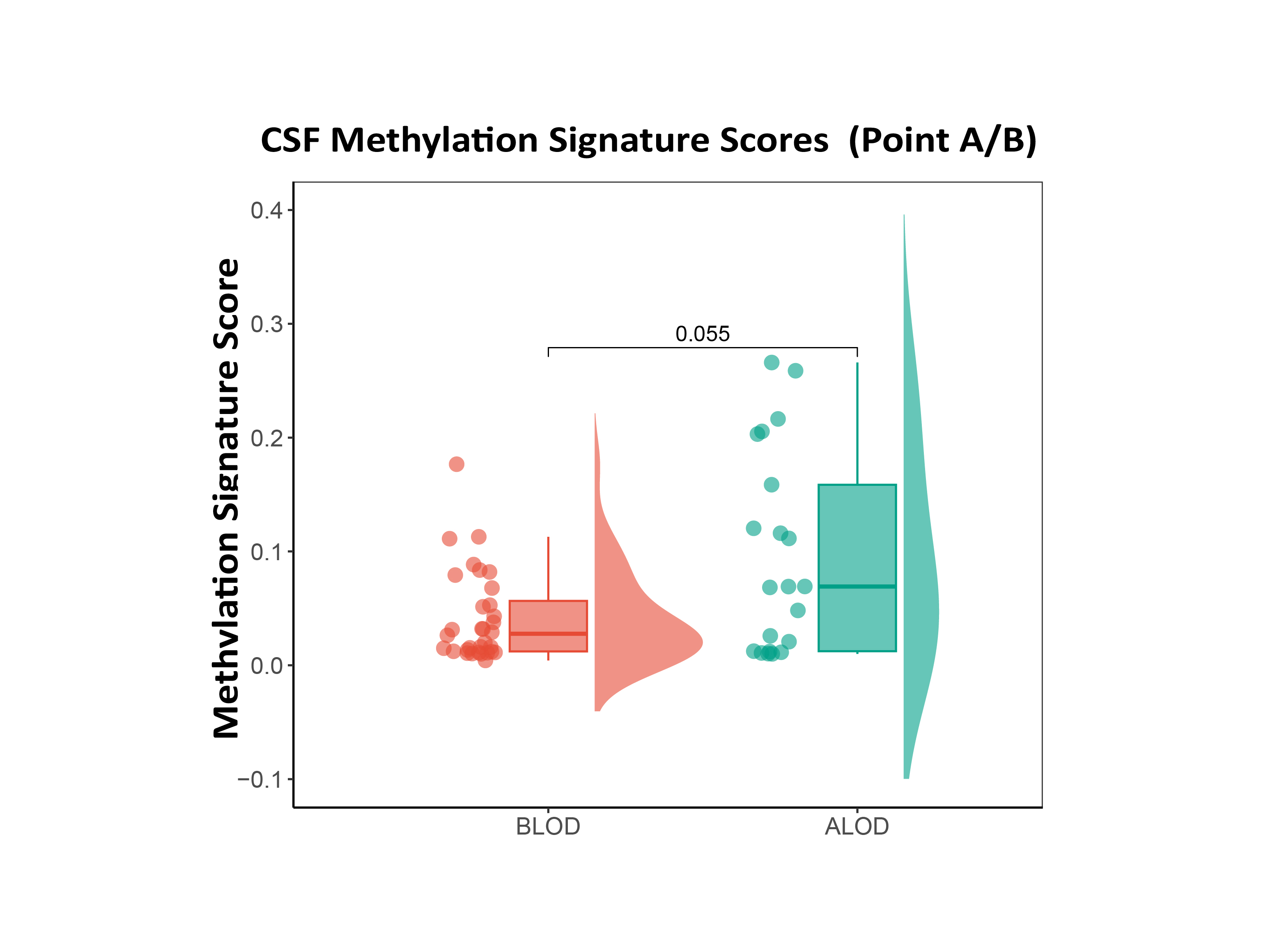


**Supplementary Figure S7.** Raincloud plot comparing Methylation Risk Score in pre-pre- and intra-operative CSF samples between BLOD and ALOD subgroups as determined by quantitative analysis of ctDNA.

**Abbreviations**: CSF, cerebrospinal fluid BLOD, below the limit of detection; ALOD, above the limit of detection.


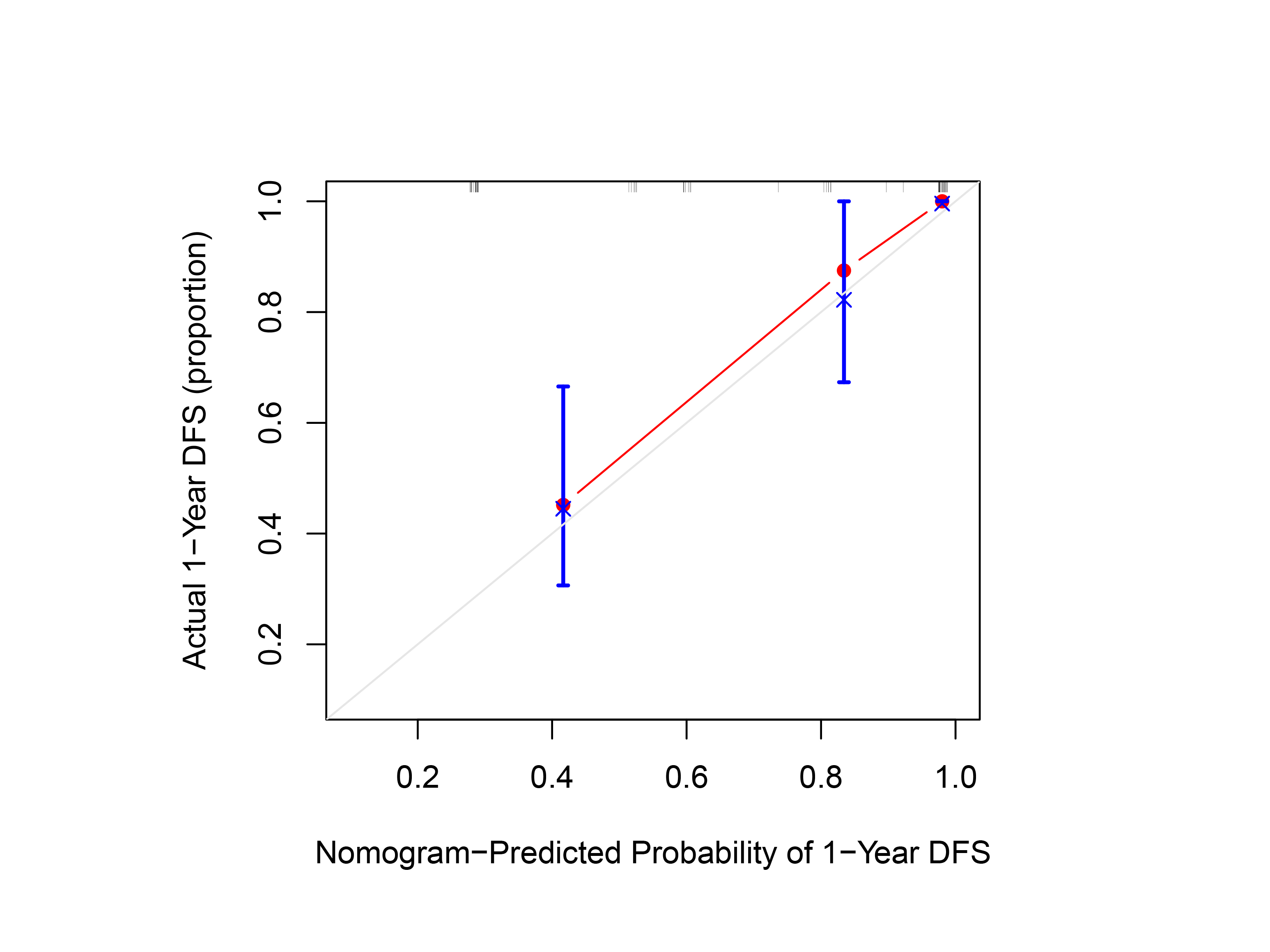


**Supplementary Figure S8.** Calibration curve to compare the predicted nomogram. The dashed diagonal line represents the ideal nomogram.


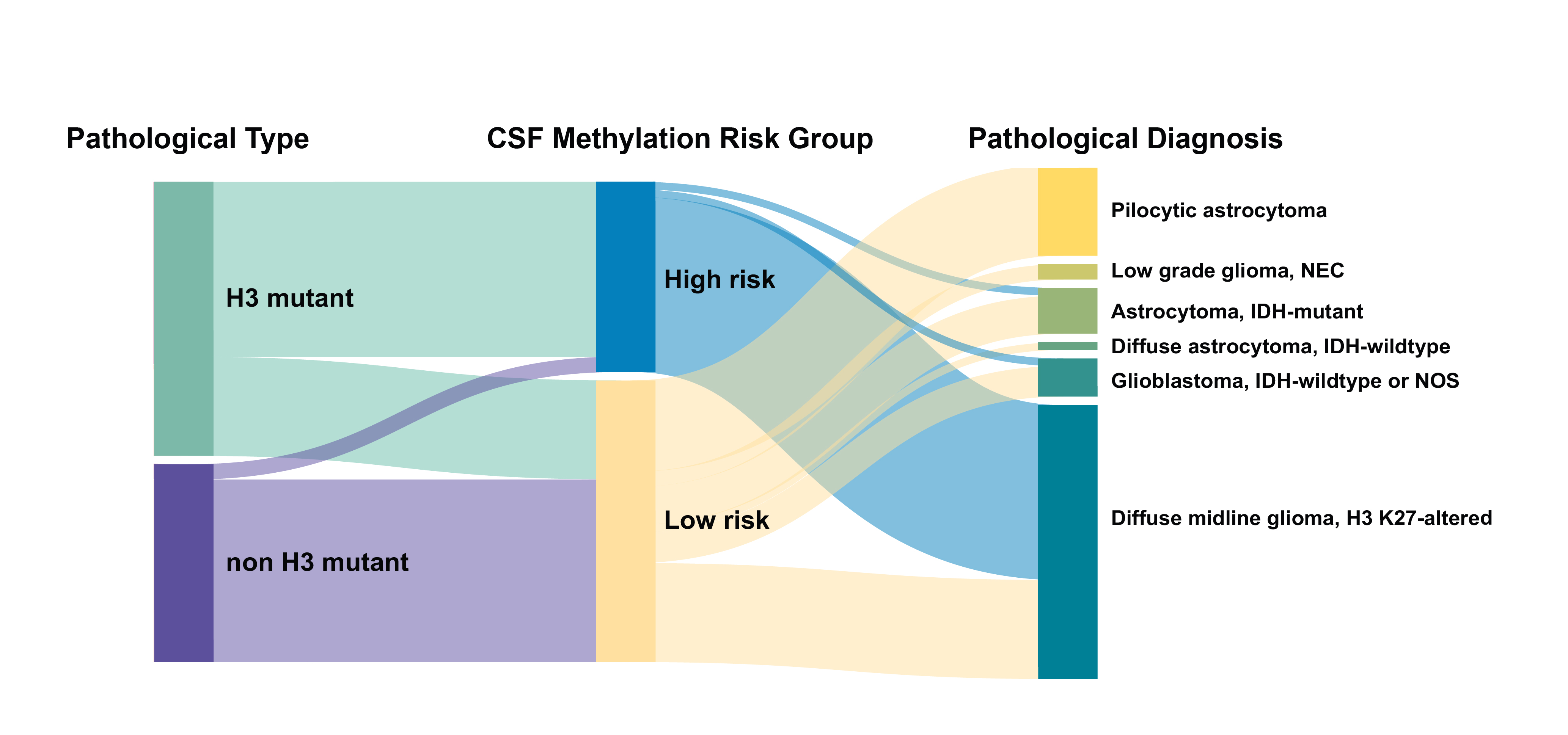


**Supplementary Figure S9.** Sankey diagram illustrating the relationships between patients' pathological type, CSF methylation group, and histopathological diagnosis.

**
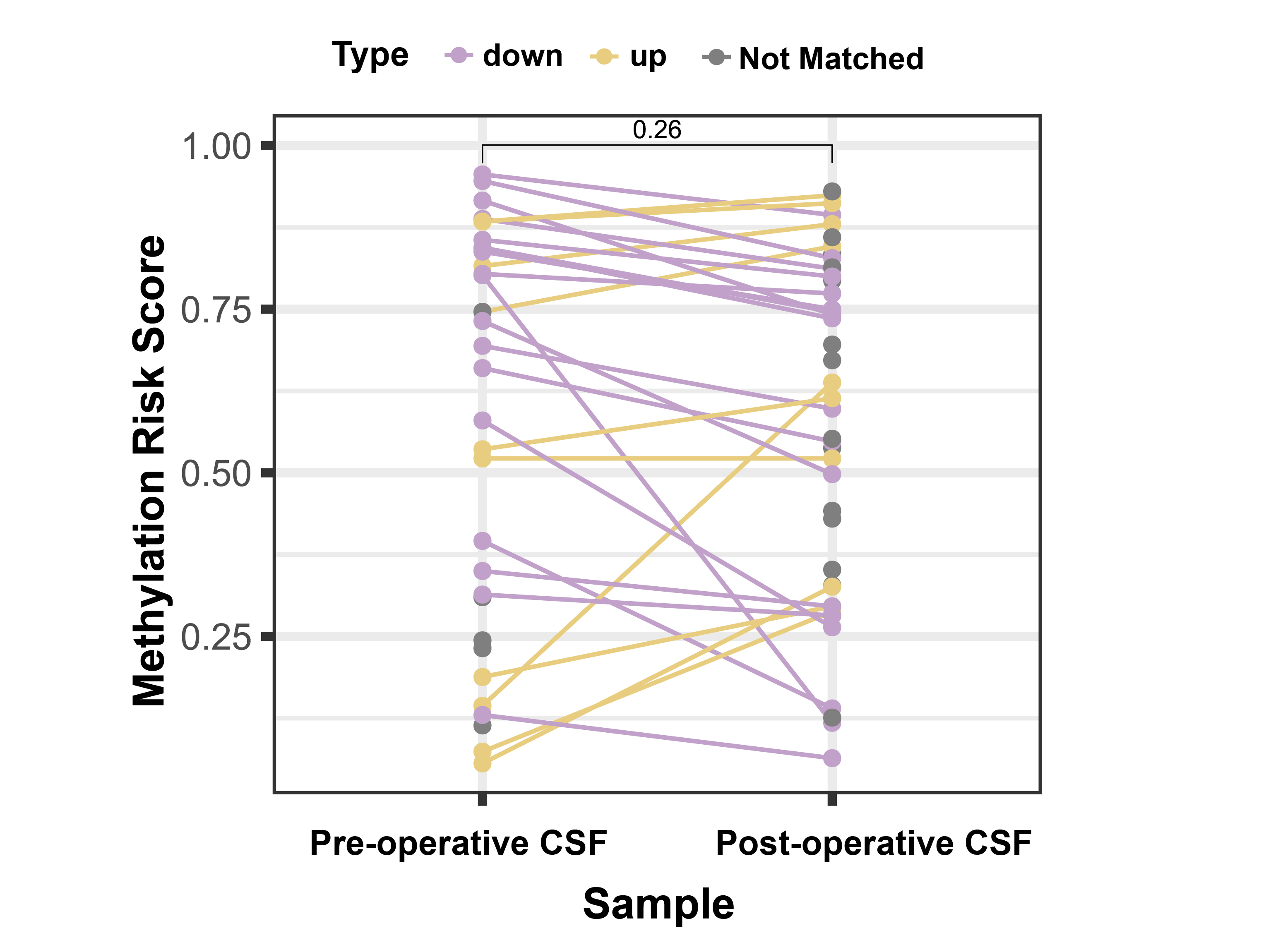
Supplementary Figure S10.** Distribution of Methylation Risk Scores (MRS) in pre-operative and post-operative CSF samples, along with paired-sample comparisons. Purple dots and connecting lines indicate patients with a postoperative decrease in MRS relative to baseline; yellow dots represent increases; grey dots denote unmatched samples.

**Abbreviations:** CSF, cerebrospinal fluid; MRS, Methylation Risk Score;

**
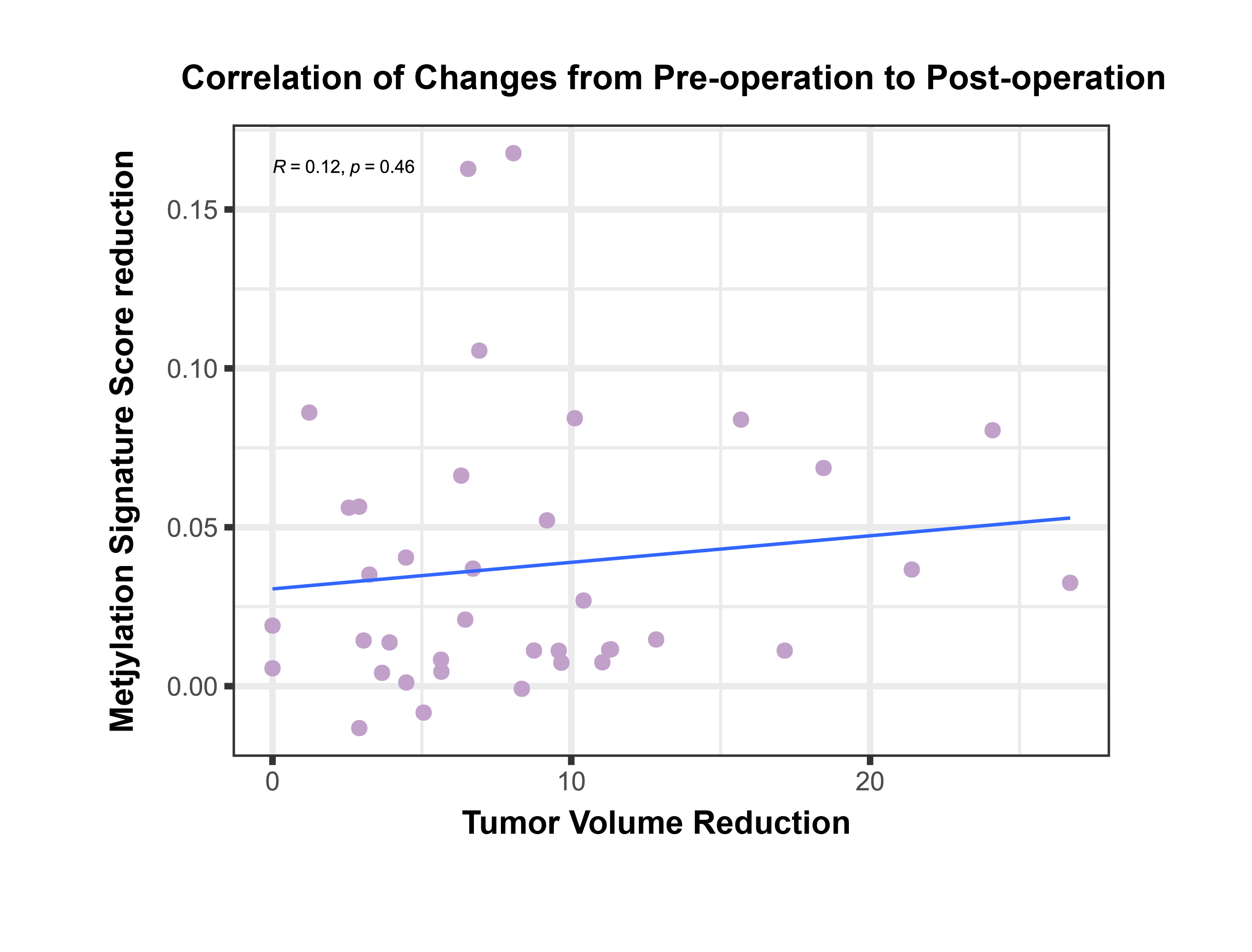
**

**Supplementary Figure S11.** Scatter plot showing the correlation of Methylation Signature Score reduction and tumor volume reduction (From pre-operation to post-operation). The X-axis indicates tumor volume reduction measured by tumor reconstruction by 3D Slicer. The Y-axis indicates the Methylation Signature Score reduction. Pearson correlation was used to assess concordance.

**Abbreviations:** VAF, Variant allele frequency; CSF, Cerebrospinal Fluid.


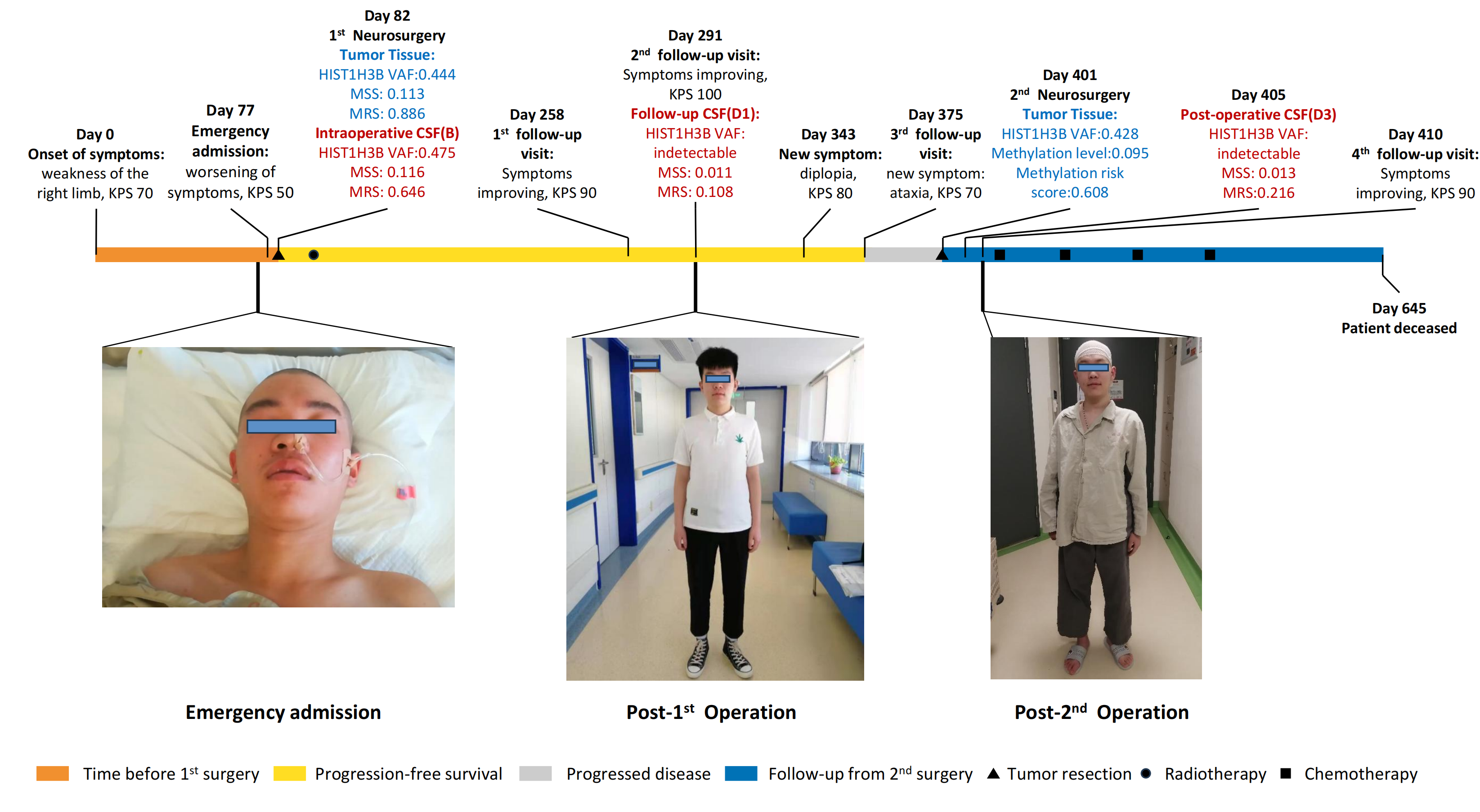


**Supplementary Figure S12.** Photographs illustrating the clinical course of Case LGN from preoperative status to the second postoperative follow-up. Improvement in the patient's clinical symptoms paralleled the reduction in tumor burden, along with a consistent decline in tissue/CSF HIST1H3B mutant VAF, Methylation Signature Scores, and Methylation Risk Scores. Together with the MRI findings shown in Fig. 6C, these results support the potential of CSF molecular profiling to reflect tumor burden and treatment response.


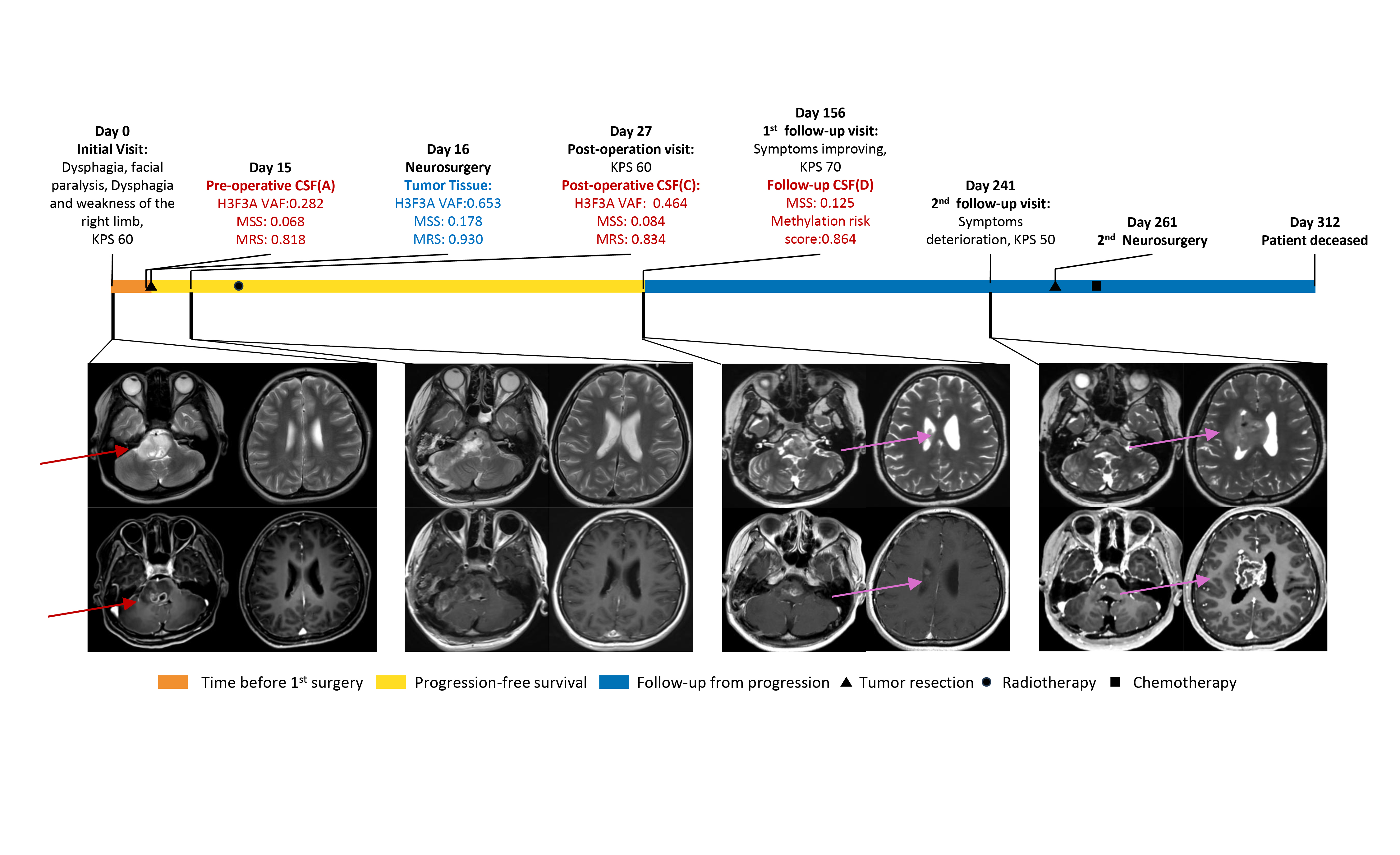


**Supplementary Figure S13.** Representative case (B137) illustrating dynamic monitoring of CSF ctDNA in patients with DMG. Colored bars represent sequential clinical phases. The four sets of MRI scans below show dynamic changes in the primary brainstem lesions (red arrows) and continuously progressed disseminated lesions in the lateral ventricles (purple arrows), from the initial preoperative assessment to the final follow-up. Annotations above the timeline indicate changes in clinical status and mutation/methylation co-detection results from tissue and/or CSF samples at each timepoint.


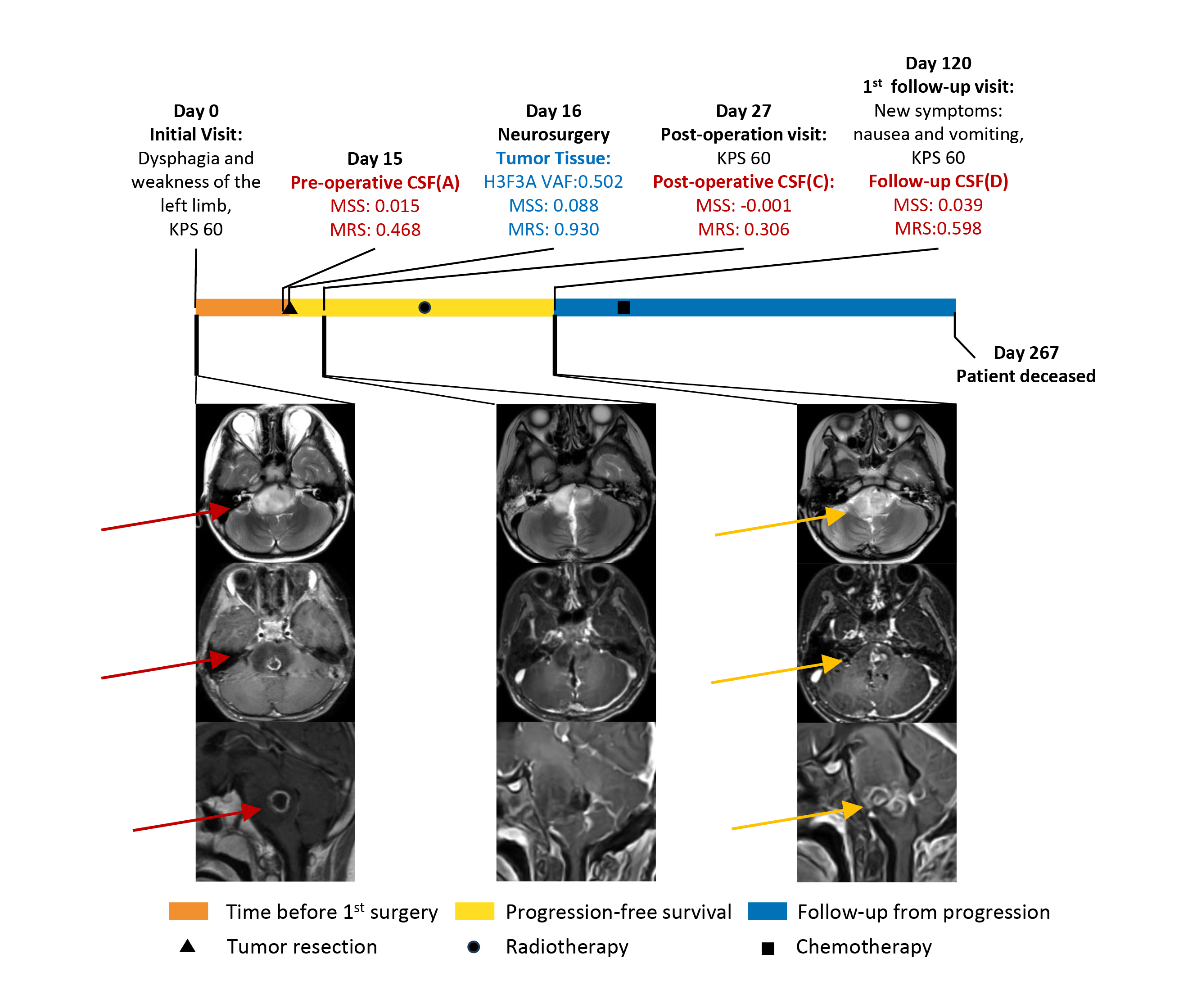


**Supplementary Figure S14.** Representative case (ZJY) illustrating dynamic monitoring of CSF ctDNA in patients with DMG. Colored bars represent sequential clinical phases. The three sets of MRI scans below demonstrate dynamic changes in the primary brainstem lesions (red arrows) and recurrent lesions at the original brainstem site (yellow arrows), from the initial preoperative assessment to the final follow-up. Annotations above the timeline indicate changes in clinical status and mutation/methylation co-detection results from tissue and/or CSF samples at each corresponding timepoint.
