# Supplementary material for: Co-detection of mutations and methylations in cerebrospinal fluid ctDNA for minimally-invasive diagnosis of brainstem glioma
Source: J Exp Clin Cancer Res. 2025 Oct 7;44:283. doi: 10.1186/s13046-025-03455-y (PMC12502398; doi:10.1186/s13046-025-03455-y)
Supplement: Supplementary file 2 — Supplementary Material 2 [file 13046_2025_3455_MOESM2_ESM.docx]

**Supplementary Methods**

**Study design and sample obtaining.**

The study was approved by the Ethics Committee of Beijing Tiantan Hospital (KY2024-139-01). All patients provided informed consent, and the study adhered to the Declaration of Helsinki. The study design is summarized in Fig. 1A. This study comprised three primary phases: 1) Screening and validating BSG-specific methylation biomarkers using public databases; 2) Training and testing a diagnostic classifier integrating methylation and mutation profiles from tissue samples; 3) Evaluating the classifier’s diagnostic efficacy in cerebrospinal fluids (CSFs). Additionally, the study explored the prognostic capabilities of methylation and monitored ctDNA dynamics throughout treatment in parallel.

The inclusion criteria for this retrospective, observational study were as follows: 1) age ≥3 years and ≤60 years; 2) diagnosed with brainstem glioma via MRI; 3) patients who underwent surgery or biopsy at Beijing Tiantan Hospital and were pathologically diagnosed with glioma; 4) patients who had at least one CSF sample collected and stored during hospitalization (≥3 ml). The exclusion criteria included: 1) patients with a history of other neurological diseases, including hemorrhagic/ischemic stroke, epilepsy, demyelinating disorders, etc; 2) patients who did not sign the informed consent form.

Thus, from 2018 to 2023, a cohort of 80 eligible BSG patients were collected: 52 cases of DMG with H3K27M mutations (H3K27M subtype), 7 cases of Astrocytoma with IDH mutations (IDH subtype), and 21 cases of H3K27M/IDH double-wildtype (DW) gliomas (DW subtypes). A total of 138 cerebrospinal fluid (CSF) samples (3-10 ml) were collected at multiple time points: 54 preoperative (Point A), 32 intraoperative (Point B), 46 postoperative (Point C), and 5 during follow-up (Point D). Additionally, 71 tissue samples were obtained (with one patient providing two tumor samples due to tumor recurrence during a second surgery), while 10 patients were excluded from sampling due to insufficient tumor tissues, prioritizing the need for pathological diagnosis. 71 peripheral blood samples were collected. Public datasets (GSE90496, GSE109379, GSE161944, GSE50022, GSE64509, EGAS00001004341) were used for the de novo design of methylation biomarkers.

**Clinical, radiological, pathological data and follow-up.**

In this study, the clinical, radiological, pathological and follow-up parameters extracted from The National Brain Tumor Registry Center of China (NBTRC), which developed and maintains a specialized registry for brainstem tumors^1,2^.

Preoperative, postoperative and follow-up MRI of all the case were obtained and re-evaluated by two experienced neurosurgeons (CC.P. and LW. Z.). Pathological diagnosis were re-reviewed by two senior pathologists, based on the 2021 WHO Classification of Central Nervous System Tumors (5th edition). Patients with glioma exhibiting positive immunohistochemical staining for H3K27M or mutations in H3F3A or HIST1H3B/C were classified as the H3K27M-mut subtype. Those with positive IDH1/2-mutant staining or sequencing were classified as the IDH-mut subtype, while all the others were defined as the Double-Wildtype (DW) subtype.

Tumor volume was calculated using 3D Slicer (v5.3.0), and the extent of tumor resection (EOR) was determined by the formula:

$$EOR=\frac{Volume\left( preoperative \right)-Volume\left( postoperative \right)}{Volume\left( preoperative \right)}$$

Follow-up data were extracted from the NBTRC, including the date of the last follow-up, survival status, and subsequent treatment plans. In this study, overall survival (OS) was the primary prognostic endpoint, defined as the duration from the date of the first MRI to the occurrence of the endpoint event or the date of the last follow-up.

**Methylation microarrays analysis.**

The Illumina HumanMethylation450 BeadChip and Infinium MethylationEPIC BeadChip were obtained from previously published reports^3-7^. IDAT files for both 450k and EPIC arrays were processed using the minfi package in R and combined into a matrix. The preprocessFunnorm function were used for normalization, and following filtering criteria were applied according to previous study^3^: removal of probes targeting the X and Y chromosomes, removal of probes containing a single-nucleotide polymorphism, and probes with poor detection P values (P > 0.01). Beta values were calculated as the ratio of the methylated signal intensity to the sum of methylated and unmethylated singles.

**Mutation and methylation assay design.**

This study utilized the Mutation Capsule technology developed in prior work, which allows for the creation of both mutation and methylation libraries^8^. DNA libraries were enriched using a specially designed brain tumor panel comprising 68 genes (GenetronHealth; Beijing, China), including major glioma-related genes, as detailed in our previous research^9^.

Since there have been no prior reports of cerebrospinal fluid ctDNA methylation detection sites specifically targeting H3K27M and IDH-mutant gliomas, this study conducted a de novo screening of methylation markers based on publicly available tissue methylation datasets.

Firstly, we accessed the 450k methylation databases for central nervous system tumors (GSE90496, GSE109379) to select methylation data from patients with pathologically diagnosed diffuse midline gliomas (DMG, N=117) and IDH-mutant gliomas (IDH, N=422), as well as from normal brain tissue (N=72). We split the data, using 70% of the cases for discovery purposes. To identify highly differentially methylated CpG loci, we set a threshold of β ≥ 0.3. Following the identification of differential loci, we employed various machine learning algorithms (Random Forest, SVM, Lasso) for feature selection**.**

Subsequently, for the dimension-reduced differential loci, we designed and validated primers based on MCP technology, identifying 76 candidate CpG markers. These markers were then tested in the remaining 30% of cases. Additionally, we selected publicly available 450k/850k methylation datasets (GSE90496, GSE161944, GSE50022, GSE64509) along with our previous dataset (EGAS00001004341) for validation. The validation set included H3K27M-mutant gliomas (N=121), IDH-mutant gliomas (N=24), and a control group comprising normal brain tissue or double-negative gliomas (N=260). Through diagnostic efficacy evaluations, including ROC analysis, we finalized the 76 differential loci as the methylation detection panel.

**The ctDNA extraction and quantification.**

DNA from CSF supernatant was isolated using Apostle MiniMax high efficiency cfDNA isolation kit (Apostle; Pleasanton, CA, USA). All concentration measurements are performed using the Qubit 3.0 Fluorometer (Life Technologies; Carlsbad, CA, USA) and Qubit dsDNA HS Assay kit (Life Technologies; Carlsbad, CA, USA) according to the recommended protocol.

**Library preparation and sequencing analysis.**

Tissue samples underwent both ctDNA mutation and methylation testing. CSF samples with <5 ng were used for methylation library construction only, while those with ≥5 ng were used for both mutation and methylation library construction. In total, 137 samples had methylation libraries established, and 86 samples had both mutation and methylation libraries established.

MCP libraries were constructed from 5 ng of DNA following established protocols. cfDNA was first digested with methylation-sensitive restriction enzymes Hha I and Hinp 1I, then ligated with customized adapters and amplified to create the pre-MCP library. 400 ng of this pre-MCP library was digested with lambda exonuclease and amplified with gene-specific primers for target regions to construct the MCP library. Additionally, 1000 ng of the pre-MCP library was captured using a brain cancer panel of 68 genes (GenetronHealth, Beijing, China).

All libraries were sequenced on an Illumina NovaSeq 6000 platform following the manufacturer's instructions. Sequencing reads were processed with a previously described bioinformatics pipeline^8,9^. For Mutation analysis, sequencing reads underwent demultiplexed and removed adapters and low-quality regions. Reads were then mapped to the hg19 reference genome using BWA (v0.7.10). PCR duplicates were marked using Picard. Pileup files were generated for the genomic regions targeted by exome enrichment. SAMtools and Pindel was used for SNV or indel calling. Additional criteria were adopted for retaining a mutation in CSF: an allele fraction of ≧0.1% and a total of ≧ 4 reads. Meanwhile, known recurrent loci were manually reviewed suing Integrative Genomics Viewer (IGV v2.16.1). For methylation analysis, sequencing reads processed as similar to mutation analysis, the molecules with a UID family containing at least 3 reads were used to calculate the degree of methylation. The sequenced molecules ending up with the GCGC restriction site were denoted as unmethylated molecules. The molecules which passed through the GCGC sequence were denoted as methylated molecules. The degree of methylation was calculated as the ratio of the number of methylated molecules to the total number of methylated and unmethylated molecules. To evaluate the overall methylation signature of the 76 CpGs, we introduced the Methylation Signature Score (MSS) by following formula:

$$Methylation Signature Score=\frac{\sum\left( H3 specific markers \right)+(1-\sum\left( IDH specific makers \right))}{Probe Number}$$

**Development of methylation classifier model in tissue samples.**

To assess whether H3K27M, IDH, and DW subtypes are distinguishable using 76 CpGs in our independent cohort, the tissue methylation profile was first validated. A heatmap was used to display the distribution of probes among the three groups. The methylation levels were then compared for differences between the three groups. Subsequently, the correlation between methylation levels and H3F3A VAF as well as IDH VAF was evaluated, with Pearson’s correlation coefficients and associated P-values calculated

In order to distinguish H3K27M, IDH and DW subtypes using methylation, a Random Forest (RF) classifier model was created to assign subgroup and corresponding probability using tissue methylation data of MCP. Model optimization was performed using tenfold cross-validation in the training cohort, with the kappa statistic serving as the summary metric for model optimization. Model performance was evaluated using independent test cohort of our previous study^7^ by sensitivity, specificity and receiver operating characteristic (ROC).

Given the association of H3K27M with poor prognosis, we introduced the Methylation Risk Score (MRS), defined as:

$$Methylation Risk Score=Predicted Probability of H3K27M in Methylation RF Models$$

**Construction and evaluation of the co-detection classifier, termed BSGdiag.**

For BSGdiag, we integrated mutation status and the methylation model (Figure 1B). The final probability for each diagnostic type is calculated as the sum of mutation-predicted and methylation-predicted probabilities. For H3K27M-mut gliomas, if an H3F3A/HIST1H3B mutation is present, the mutation-predicted probability is 1, otherwise 0. For IDH-mut gliomas, an IDH1/IDH2 mutation gives a mutation-predicted probability of 1. For the double-wildtype, all mutation-predicted probabilities are 0. Methylation-predicted probabilities are obtained via aforementioned methylation classifier model. The definitive diagnosis is ascertained based on the subtype that the BSGdiag model identifies as having the highest probability. The dataset from our previous study^7^ serves as an independent testing set for model evaluation.

**Validation of mutation detection in CSF**

We employed a waterfall plot to illustrate the mutation frequencies in cerebrospinal fluid (CSF) and to depict the types of mutations and their frequencies. Furthermore, we assessed the correlation between tissue and CSF H3F3A mutation variant allele frequencies (VAFs) by calculating Pearson’s correlation coefficients and associated P-values

**Validation of selected methylation markers in CSF**

We used a heatmap to display the methylation profiles in the cerebrospinal fluid (CSF) cohort and evaluated the inter-group differences in CSF methylation levels among the H3K27M, IDH, and DW groups. Additionally, Pearson correlation tests were performed to validate the correlation between CSF methylation levels and H3F3A mutation VAF, as well as the correlation between CSF methylation levels and tissue methylation levels.

Additionally, we further assessed whether ctDNA content affects methylation detection by comparing the differences in CSF methylation levels between the ALOD and BLOD groups, as well as the differences in the CSF methylation risk score obtained from RF model between the two groups.

**Evaluation of diagnostic accuracy of co-detection classifier in CSF**

To better reflect clinical practice, we included cerebrospinal fluid samples from points A and B in the diagnostic cohort, comprising 46 H3K27M-mut subtype, 8 IDH-mut subtype, and 22 double-wildtype subtype cases.

We first assessed the diagnostic performance of the methylation classifier model on samples that completed methylation testing, evaluating sensitivity, specificity, AUC (Area Under the Curve), macro-AUC, and micro-AUC. Macro-AUC represents the average AUC calculated for each class individually, while micro-AUC averages AUC across all classes. The 95% confidence intervals (CI) for AUC and sensitivity and specificity were estimated separately using bootstrapping and Agresti-Coull method. which involve repeatedly sampling the dataset with replacement to assess variability and confidence**.**

Subsequently, Samples that underwent both mutation and methylation testing (23 H3K27M-mut subtype, 2 IDH-mut subtype, and 4 DW-subtype cases) were utilized to access the diagnostic performance of the BSGdiag model.

**Assessment of risk stratification of methylation features.**

For the prognosis study, we included patients who completed methylation testing at points A and B and had H3K27M mutation status (in tissue or cerebrospinal fluid), resulting in 41 H3K27M mutants, 7 IDH mutants, and 21 double-wildtype cases.

The Methylation Risk Score cut-off was determined using the R package 'survminer' to optimize prognostic differentiation. Based on this cut-off, patients were classified into high and low methylation groups. Kaplan-Meier curves illustrated prognosis differences, with the log-rank test assessing statistical significance.

Univariate Cox regression was used to identify prognostic factors."Noninvasive prognostic variables included sex, age, imaging features such as tumor group, involvement of the midbrain, pons, and medulla, infiltration, and contrast enhancement, as well as CSF-accessible factors such as pathological subtype ( H3K27M mutation status) and CSF risk group stratified by the MRS-based cut-off. Variables with p < 0.05 were included in multivariate Cox regression were used to identify independent prognostic factors. A nomogram based on the multivariate Cox model was developed for prognosis prediction, and model performance was evaluated using calibration plots and the Hosmer-Lemeshow test. Independent prognostic factors were visualized with a Sankey diagram.

**Evaluation of dynamic monitoring potential of CSF mutation and methylation detection.**

This study performed dynamic point testing for DMG patients, including 44 patients at point C and 3 at point D, with one patient providing samples from two time points, resulting in 4 samples at point D. Paired sample analysis was utilized to compare preoperative and postoperative methylation levels and methylation risk scores. Pearson correlation analysis evaluated the relationship between tumor resection volume and changes in these indicators.

**Statistical Analysis**

For continuous variables consistent with normal distribution, the independent sample t-test and one-way ANOVA were used, while for continuous variables with non-normal distribution, the Mann-Whitney U-test and Kruskal-Wallis test were used. The Wilcoxon signed-rank test was used for paired samples in two groups, and the Friedman test was used for paired samples in multiple groups. For categorical variables, the chi-square test or Fisher exact probability method was used. Correlation analyses were performed using the Pearson correlation coefficient.

Sensitivity and specificity were calculated using the following formulas: sensitivity = [true positives / (true positives + false negatives)] × 100%, and specificity = [true negatives / (true negatives + false positives)] × 100%. The 95% confidence intervals (CIs) for sensitivity and specificity were estimated using the Agresti-Coull method. Receiver operating characteristic (ROC) curves were constructed to assess diagnostic performance, and the area under the curve (AUC) along with its 95% CI was estimated using nonparametric bootstrapping (R = 100 iterations) with equi-tailed two-sided intervals to assess diagnostic performance. For survival analysis, both univariate and multivariate Cox proportional hazards regression hazard regression analysis were employed.

Those *P*-values with two-tailed *P* < .05 were considered statistically significant. All graphs and statistical analyses were done using R software (4.3.2). For key analyses these include: ‘Complexheatmap’, ‘caret’, ‘multiROC’, ‘ggplot2’, ‘gghalves’, ‘ggpubr’, ‘dplyr’, ‘stats’, ‘survival’, ‘survminer’, ‘compareGroups’. ‘tidyverse’, ‘rms’ and ‘pheatmap’.

**Data and Materials Availability**

All data generated in this manuscript are publicly available, access requires a request to the corresponding authors in accordance with institutional policies. Data was deposit in the China National Center for Bioinformation (CNCB) and assigned the ID subHRA012094.

**References**

1. Xiao D, Yan C, Li D, et al. National Brain Tumour Registry of China (NBTRC) statistical report of primary brain tumours diagnosed in China in years 2019-2020. *The Lancet regional health Western Pacific*. May 2023;34:100715. doi:10.1016/j.lanwpc.2023.100715

2. Zhang L, Jia W, Ji N, et al. Construction of the National Brain Tumor Registry of China for better management and more efficient use of data: a protocol. *BMJ open*. Jan 17 2021;11(1):e040055. doi:10.1136/bmjopen-2020-040055

3. Capper D, Jones DTW, Sill M, et al. DNA methylation-based classification of central nervous system tumours. *Nature*. Mar 22 2018;555(7697):469-474. doi:10.1038/nature26000

4. Schüller U, Iglauer P, Dorostkar MM, et al. Mutations within FGFR1 are associated with superior outcome in a series of 83 diffuse midline gliomas with H3F3A K27M mutations. *Acta neuropathologica*. Feb 2021;141(2):323-325. doi:10.1007/s00401-020-02259-y

5. Buczkowicz P, Hoeman C, Rakopoulos P, et al. Genomic analysis of diffuse intrinsic pontine gliomas identifies three molecular subgroups and recurrent activating ACVR1 mutations. *Nature genetics*. May 2014;46(5):451-6. doi:10.1038/ng.2936

6. Horvath S, Mah V, Lu AT, et al. The cerebellum ages slowly according to the epigenetic clock. *Aging*. May 2015;7(5):294-306. doi:10.18632/aging.100742

7. Chen LH, Pan C, Diplas BH, et al. The integrated genomic and epigenomic landscape of brainstem glioma. *Nature communications*. Jun 17 2020;11(1):3077. doi:10.1038/s41467-020-16682-y

8. Wang P, Song Q, Ren J, et al. Simultaneous analysis of mutations and methylations in circulating cell-free DNA for hepatocellular carcinoma detection. *Science translational medicine*. Nov 23 2022;14(672):eabp8704. doi:10.1126/scitranslmed.abp8704

9. Pan C, Diplas BH, Chen X, et al. Molecular profiling of tumors of the brainstem by sequencing of CSF-derived circulating tumor DNA. *Acta neuropathologica*. Feb 2019;137(2):297-306. doi:10.1007/s00401-018-1936-6
